# Supplementary material for: Improvement of growth, lipid metabolism and innate immune response in Pacific white shrimp (Penaeus vannamei) post-larvae through enrichment of live feeds with Schizochytrium sp., taurine and inosine monophosphate
Source: PLoS One. 2026 Mar 5;21(3):e0341999. doi: 10.1371/journal.pone.0341999 (PMC12962509; doi:10.1371/journal.pone.0341999)
Supplement: S1 Fig — Photographs were taken to assess growth performance. No abnormal signs, such as shell discoloration, hepatopancreas color changes, melanization, body-wall damage, deformities, or abnormal pigmentation, were observed. (DOCX) [file pone.0341999.s001.docx]

**Supporting Information Fig S1.** Measurement of total body length of Pacific white shrimp (*Penaeus vannamei*) post-larvae at the end of the feeding trials. Photographs were taken to assess growth performance. No abnormal signs, such as shell discoloration, hepatopancreas color changes, melanization, body-wall damage, deformities, or abnormal pigmentation, were observed.
