# Supplementary material for: Improvement of growth, lipid metabolism and innate immune response in Pacific white shrimp (Penaeus vannamei) post-larvae through enrichment of live feeds with Schizochytrium sp., taurine and inosine monophosphate
Source: PLoS One. 2026 Mar 5;21(3):e0341999. doi: 10.1371/journal.pone.0341999 (PMC12962509; doi:10.1371/journal.pone.0341999)
Supplement: Attachment — Submitted filename: PONE-D-25-53314_Rev.pdf [file pone.0341999.s003.pdf]

PLOS ONE

Improvement of growth, lipid metabolism and innate immune response in Pacific white shrimp (Penaeus vannamei) post-larvae through enrichment of zoo-live feeds with Schizochytrium sp., taurine and inosine monophosphate

--Manuscript Draft--

**Manuscript Number:**	PONE-D-25-53314
**Article Type:**	Research Article
**Full Title:**	Improvement of growth, lipid metabolism and innate immune response in Pacific white shrimp (Penaeus vannamei) post-larvae through enrichment of zoo-live feeds with Schizochytrium sp., taurine and inosine monophosphate
**Short Title:**	Functional enrichment of zoo-live feeds for Penaeus vannamei
**Corresponding Author:**	Kyeong-Jun Lee, Ph.D<br>Jeju National University<br>Jeju-si, Jeju KOREA, REPUBLIC OF
**Keywords:**	Inosine monophosphate; Penaeus vannamei; Schizochytrium; Taurine; Zoo-live feeds
**Abstract:**	The effects of zoo-live feeds enriched with Schizochytrium sp. (SCH), taurine and inosine monophosphate (IMP) on the post-larvae (PL) stages of Penaeus vannamei were investigated. In the first trial, shrimp at the PL1-2 stage were fed enriched rotifers for 9 days. The control group (RN) provided rotifers that were not enriched with nutrients. The experimental groups were provided with rotifers enriched with 0.5% SCH (RS), RS+0.1% taurine (RS+T), RS+0.1% IMP (RS+I) or 0.5% Chlorella vulgaris powder (RCL). Growth was significantly higher in RS, RS+T and RS+I groups compared to RN and RCL groups. Survival was significantly higher in RS, RS+T and RS+I groups than in RN group. Whole-body DHA levels were significantly higher in the RS, RS+T and RS+I groups than in RN and RCL groups. Relative gene expressions levels of FAS, FABP, FATP,CPT1, lysozyme, crustin, LGBP, Pen-3a and proPO were significantly upregulated in all SCH-enriched groups compared to RN group. In the second trial, shrimp at the PL12-14 stage were fed enriched Artemia for 12 days. The control group (AN) received an unenriched Artemia. The experimental groups were provided with Artemia enriched with 0.5% SCH (AS), AS+0.1% taurine (AS+T), AS+0.1% IMP (AS+I) and 0.5% Chlorella powder (ACL). Growth performance was significantly higher in the SCH-enriched groups compared to AN and ACL groups whereas, survival did not differ significantly among the experimental groups. Whole-body DHA levels were significantly higher in the AS, AS+T and AS+I groups than in AN and ACL groups. Relative gene expressions levels of FAS, FABP, FATP,CPT1, lysozyme, crustin, LGBP, Pen-3a and proPO were significantly upregulated in all SCH-enriched groups compared to AN group. This study demonstrated that supplementation of live feeds with SCH effectively enhanced shrimp growth, lipid metabolism, immunity and survival, and that combined enrichment with taurine and IMP provided even greater immunostimulatory effects.
**Order of Authors:**	Suhyeok Kim
	Sanghyun Song
	Yeonji Lee
	Yein Lee
	Young-Mi Lee
	Kook-Jin Cho
	Kyeong-Jun Lee, Ph.D
**Additional Information:**	
**Question**	**Response**
**Financial Disclosure**	Yes

Powered by Editorial Manager® and ProduXion Manager® from Aries Systems Corporation

Enter a financial disclosure statement that describes the sources of funding for the work included in this submission. Review the submission guidelines for detailed requirements. View published research articles from *PLOS ONE* for specific examples.<br><br>This statement is required for submission and **will appear in the published article** if the submission is accepted. Please make sure it is accurate.<br><br>**Funded studies**<br>Enter a statement with the following details:<br>• Initials of the authors who received each award<br>• Grant numbers awarded to each author<br>• The full name of each funder<br>• URL of each funder website<br>• Did the sponsors or funders play any role in the study design, data collection and analysis, decision to publish, or preparation of the manuscript?<br><br>Did you receive funding for this work?	
Please add funding details.<br>as follow-up to "**Financial Disclosure**<br><br>Enter a financial disclosure statement that describes the sources of funding for the work included in this submission. Review the submission guidelines for detailed requirements. View published research articles from *PLOS ONE* for specific examples.<br><br>This statement is required for submission and **will appear in the published article** if the submission is accepted. Please make sure it is accurate.	This work was supported by the CJ Cheiljedang Co., Ltd and Basic Science Research Program through the National Research Foundation of Korea (NRF) funded by the Ministry of Education (RS-2019-NR040078)

Powered by Editorial Manager® and ProduXion Manager® from Aries Systems Corporation

**Funded studies**<br>Enter a statement with the following details:<br>• Initials of the authors who received each award<br>• Grant numbers awarded to each author<br>• The full name of each funder<br>• URL of each funder website<br>• Did the sponsors or funders play any role in the study design, data collection and analysis, decision to publish, or preparation of the manuscript?<br><br>Did you receive funding for this work?"	
Please select the country of your main research funder (please select carefully as in some cases this is used in fee calculation).<br>as follow-up to "**Financial Disclosure**<br><br>Enter a financial disclosure statement that describes the sources of funding for the work included in this submission. Review the submission guidelines for detailed requirements. View published research articles from *PLOS ONE* for specific examples.<br><br>This statement is required for submission and **will appear in the published article** if the submission is accepted. Please make sure it is accurate.<br><br>**Funded studies**<br>Enter a statement with the following details:<br>• Initials of the authors who received each award<br>• Grant numbers awarded to each author<br>• The full name of each funder<br>• URL of each funder website<br>• Did the sponsors or funders play any role in the study design, data collection and analysis, decision to publish, or preparation of the manuscript?<br><br>Did you receive funding for this work?"	KOREA, REPUBLIC OF - KR
**Competing Interests**	The authors declare that they have no known competing financial interests or personal

Use the instructions below to enter a competing interest statement for this submission. On behalf of all authors, disclose any competing interests that could be perceived to bias this work—acknowledging all financial support and any other relevant financial or non-financial competing interests.<br><br>This statement is required for submission and **will appear in the published article** if the submission is accepted. Please make sure it is accurate and that any funding sources listed in your Funding Information later in the submission form are also declared in your Financial Disclosure statement.<br><br>View published research articles from *PLOS ONE* for specific examples.<br><br>**NO authors have competing interests**<br><br>Enter: *The authors have declared that no competing interests exist.*<br><br>**Authors with competing interests**<br><br>Enter competing interest details beginning with this statement:<br><br>*I have read the journal's policy and the authors of this manuscript have the following competing interests: [insert competing interests here]*<br><br>* typeset	relationships that could have appeared to influence the work reported in this paper.
**Ethics Statement**<br><br>Enter an ethics statement for this submission. This statement is required if the study involved:<br><br>• Human participants<br>• Human specimens or tissue<br>• Vertebrate animals or cephalopods<br>• Vertebrate embryos or tissues	N/A

• Field research<br><br>Write "N/A" if the submission does not require an ethics statement.<br><br>General guidance is provided below. Consult the submission guidelines for detailed instructions. **Make sure that all information entered here is included in the Methods section of the manuscript.**	

Powered by Editorial Manager® and ProduXion Manager® from Aries Systems Corporation

**Format for specific study types**<br><br>**Human Subject Research (involving human participants and/or tissue)**<br>• Give the name of the institutional review board or ethics committee that approved the study<br>• Include the approval number and/or a statement indicating approval of this research<br>• Indicate the form of consent obtained (written/oral) or the reason that consent was not obtained (e.g. the data were analyzed anonymously)<br><br>**Animal Research (involving vertebrate animals, embryos or tissues)**<br>• Provide the name of the Institutional Animal Care and Use Committee (IACUC) or other relevant ethics board that reviewed the study protocol, and indicate whether they approved this research or granted a formal waiver of ethical approval<br>• Include an approval number if one was obtained<br>• If the study involved *non-human primates*, add *additional details* about animal welfare and steps taken to ameliorate suffering<br>• If anesthesia, euthanasia, or any kind of animal sacrifice is part of the study, include briefly which substances and/or methods were applied<br><br>**Field Research**<br><br>Include the following details if this study involves the collection of plant, animal, or other materials from a natural setting:<br>• Field permit number<br>• Name of the institution or relevant body that granted permission	
**Data Availability**<br><br>Authors are required to make all data underlying the findings described fully available, without restriction, and from the time of publication. PLOS allows rare exceptions to address legal and ethical concerns. See the PLOS Data Policy and FAQ for detailed information.	Yes - all data are fully available without restriction

Powered by Editorial Manager® and ProduXion Manager® from Aries Systems Corporation

A Data Availability Statement describing where the data can be found is required at submission. Your answers to this question constitute the Data Availability Statement and **will be published in the article**, if accepted.<br><br>**Important:** Stating 'data available on request from the author' is not sufficient. If your data are only available upon request, select 'No' for the first question and explain your exceptional situation in the text box.<br><br>Do the authors confirm that all data underlying the findings described in their manuscript are fully available without restriction?	
**Describe where the data may be found in full sentences. If you are copying our sample text, replace any instances of XXX with the appropriate details.**<br><br>• If the data are **held or will be held in a public repository**, include URLs, accession numbers or DOIs. If this information will only be available after acceptance, indicate this by ticking the box below. For example: *All XXX files are available from the XXX database (accession number(s) XXX, XXX.)*.<br>• If the data are all contained **within the manuscript and/or Supporting Information files**, enter the following: *All relevant data are within the manuscript and its Supporting Information files.*<br>• If neither of these applies but you are able to provide **details of access elsewhere**, with or without limitations, please do so. For example:<br><br>*Data cannot be shared publicly because of [XXX]. Data are available from the XXX Institutional Data Access / Ethics Committee (contact via XXX) for researchers who meet the criteria for access to confidential data.*<br><br>*The data underlying the results presented in the study are available from (include the name of the third party*	All relevant data are within the paper.

and contact information or URL).<br>• This text is appropriate if the data are owned by a third party and authors do not have permission to share the data.<br><br>* typeset	
Additional data availability information:	

Manuscript

Click here to access/download;Manuscript;[MS] CJMA_Rotifer and Artemia.docx

Improvement of growth, lipid metabolism and innate immune response in Pacific white shrimp (*Penaeus vannamei*) post-larvae through enrichment of zoo-live feeds with *Schizochytrium* sp., taurine and inosine monophosphate

Suhyeok Kim[1], Sanghyun Song[1], Yeonji Lee[1], Yein Lee[1], Young-Mi Lee[2], Kook-Jin Cho[2] and Kyeong-Jun Lee[1,3] *

[1]Department of Marine Life Sciences, Jeju National University, Jeju 63243, South Korea

[2]CJ Cheiljedang Co., Seoul 04560, South Korea

[3]Marine Life Research Institute, Jeju National University, Jeju 63333, South Korea

*Corresponding author: Kyeong-Jun Lee

E-mail address: kjlee@jejunu.ac.kr

Tel: +82-64-754-3423

Fax: +82-64-756-3493

Short title: Functional enrichment of zoo-live feeds for *P. vannamei*

Abstract

The effects of zoo-live feeds enriched with *Schizochytrium* sp. (SCH), taurine and inosine monophosphate (IMP) on the post-larvae (PL) stages of *Penaeus vannamei* were investigated. In the first trial, shrimp at the PL1-2 stage were fed enriched rotifers for 9 days. The control group (RN) provided rotifers that were not enriched with nutrients. The experimental groups were provided with rotifers enriched with 0.5% SCH (RS), RS+0.1% taurine (RS+T), RS+0.1% IMP (RS+I) o[illegible]5% *Chlorella vulgaris* powder (RCL). Growth was significantly higher in RS, RS+T and RS+I groups compared to RN and RCL groups. Survival was significantly higher in RS, RS+T and RS+I groups than in RN group. Whole-body DHA levels were significantly higher in the RS, RS+T and RS+I groups than in RN and RCL groups. Relative gene expressions levels of *FAS*, *FABP*, *FATP*,*CPT1*, *lysozyme*, *crustin*, *LGBP*, *Pen-3a* and *proPO* were significantly upregulated in all SCH-enriched groups compared to RN group. In the second trial, shrimp at the PL12-14 stage were fed enriched *Artemia* for 12 days. The control group (AN) received an unenriched *Artemia*. The experimental groups were provided with *Artemia* enriched with 0.5% SCH (AS), AS+0.1% taurine (AS+T), AS+0.1% IMP (AS+I) and 0.5% *Chlorella* powder (ACL). Growth performance was significantly higher in the SCH-enriched groups compared to AN and ACL groups whereas, survival did not differ significantly among the experimental groups. Whole-body DHA levels were significantly higher in the AS, AS+T and AS+I groups than in AN and ACL groups. Relative gene expressions levels of *FAS*, *FABP*, *FATP*,*CPT1*, *lysozyme*, *crustin*, *LGBP*, *Pen-3a* and *proPO* were significantly upregulated in all SCH-enriched groups compared to AN group. This study demonstrated that supplementation of live feeds with SCH effectively enhanced shrimp growth, lipid metabolism, immunity and survival, and that combined enrichment with taurine and IMP provided even greater immunostimulatory effects.

Key words: Inosine monophosphate, *Penaeus vannamei*, *Schizochytrium*, Taurine, Zoo-live feeds

Introduction

Pacific white shrimp (*Penaeus vannamei*) is the most widely farmed shrimp species globally, with production steadily increasing [1]. Shrimp undergo several larval stages, including nauplius, zoea, mysis and post-larvae (PL) [2]. In shrimp hatcheries, the quality of live feeds is crucial for normal development and optimal growth of the shrimp PL. Rotifer (*Brachionus plicatilis*) and *Artemia* are the most commonly used zoo-live feeds [3], because they are highly digestible and can stimulate feeding responses of larvae without adverse effect on the culture water quality [4]. However, they lack essential nutrients such as essential fatty acids, amino acids and vitamins [5]. When the rotifers and *Artemia* are used as larval feeds, their nutritional enrichment is usually needed to ensure that the larvae can receive the required essential nutrients [3]. Microalgae have been widely recognized as the most suitable source of the nutritional enrichment for the live feeds [5]. They are easy to cultivate and can be produced in large quantities, with high protein (30–50%) and lipid (10–20%) levels as well as an abundance of vitamins and carotenoids, making them highly valuable for the larval culture [6].

Schizochytrium sp. (SCH), a type of microalgae, has gained considerable attention as a potential substitute for fish oil and a functional additive in diets for Atlantic salmon (*Salmo salar*) and Pacific white shrimp due to its high level of docosahexaenoic acid (DHA, C22:6n3) [7, 8]. SCH is also rich in astaxanthin, niacin and pyridoxine, which play important roles in antioxidant capacity, immunity and disease resistance of aquatic animals [5]. An enrichment of *Artemia* with SCH was reported to improve growth, stress resistance and survival of turbot (*Scophthalmus maximus*) [9] and silver pompano (*Trachinotus blochii*) [10]. However, to the best of our knowledge, there has been extremely limited research on crustaceans including Pacific white shrimp. Another microalgae, *Chlorella* sp. is commonly and widely used as a nutritional enrichment source for the live feeds because of its low cost and easiness of cultivation [11]. *Chlorella*, however, is deficient in DHA which is one of the most important fatty acids for fish or shrimp larval growth [12]. Although DHA can be synthesized from dietary α-linolenic acid (C18:3n3) through sequential reactions catalyzed by Δ4/Δ6 desaturases and

elongases of very long-chain fatty acids 5, leading to the formation of eicosapentaenoic acid (EPA, C20:5n3) and docosapentaenoic acid (DPA, C22:5n3), or alternatively via the Sprecher pathway from EPA through elongation, Δ6 desaturation and β-oxidation, the endogenous production is insufficient to fully support optimal growth [13, 14]. Thus, a supplemental dietary DHA intake is crucial to optimize and/or maximize the growth and survival of the shrimp PL [14]. To address this concern, two consecutive studies were conducted to examine the potential application of SCH as a DHA source through the nutrition enrichment of the rotifers (the first experiment, EXP-1) and *Artemia* (the second experiment, EXP-2).

Taurine plays an important role in osmoregulation, bile salt formation and modulating cellular processes like oxidative stress and immune responses [15]. In aquatic animals, taurine has been shown to improve growth performance and stress resistance by stabilizing cellular membranes and promoting the immune functions [16]. Inosine monophosphate (IMP) is a purine nucleotide involved in cellular energy metabolism, serving as a precursor for adenosine triphosphate synthesis [17]. IMP plays a role in the enhancement of growth, stress resistance and survival by supporting mitochondrial function and improving cellular energy reserves [18]. These two supplements were expected to improve not only the nutritional quality of the zoo-live feeds but also the shrimp immunity and lipid metabolism. To date, limited attention has been given to the application of SCH as an enrichment source for live feeds in crustaceans, particularly in Pacific white shrimp post-larvae. Furthermore, no study has simultaneously evaluated the combined effects of SCH, taurine and IMP on growth, survival, and the molecular responses related to immunity and lipid metabolism. Therefore, this study represents a novel attempt to establish a multifunctional enrichment strategy that extends beyond conventional microalgal supplementation and provides mechanistic insights into shrimp larval physiology.

Materials and methods

Schizochytrium powder

SCH was provided by CJ CheilJedang (Seoul, South Korea). The SCH strain used in this study was isolated from the South Sea, South Korea and cultured in GYPS medium comprising 20 g/L D-glucose, 2 g/L yeast extract, 6 g/L peptone, 17 g/L sucrose, 12 g/L sea salt and 15 g/L bacto-agar. The strain was characterized and deposited at the Korea Collection for Type Cultures (KCTC) under the accession number KCTC15006BP. For scale-up production, the microalgae were cultivated in a 3,000 L fermenter with glucose supplementation. The fermentation broth was subsequently processed in a granulator to produce dried biomass. The proximate composition and fatty acid profile of all nutritional enrichment for zoo-live feed are shown in Table 1.

Table 1. Proximate composition and fatty acid profiles of enrichment sources.

	CP	SCH	SCHT	SCHI
Proximate composition (%, dry matter)				
Crude protein	63.0	70.5	70.6	70.5
Crude lipid	7.18	20.1	20.1	20.1
Crude ash	7.07	8.37	8.14	8.47
Fatty acid (%, lipid)				
C14	ND	2.28	2.27	2.28
C16	20.1	52.9	51.9	53.1
C17:1	20.5	ND	ND	ND
C18	ND	2.29	2.30	2.28
C18:1n9c	5.28	ND	ND	ND
C18:2n6c	50.3	ND	ND	ND
C18:3n3	3.84	ND	ND	ND
C22:6n3	ND	42.6	43.5	42.3
∑SFA[1]	20.1	57.5	56.5	57.7
∑MUFA[2]	25.8	ND	ND	ND
∑PUFA[3]	54.1	42.6	43.5	42.3

Abbreviations: CP, *Chlorella vulgaris* powder; SCH, *Schizochytrium*; SCHT, *Schizochytrium* added with 0.1% taurine; SCHI, *Schizochytrium* added with 0.1% inosine monophosphate; ND, not detected.

[1]The sum saturated fatty acids
[2]The sum monounsaturated fatty acids
[3]The sum polyunsaturated fatty acids

Rotifers culture and enrichment

Rotifers were stocked in a tank (50 L) at a density of 5,000-10,000 individuals/mL and fed with *Chlorella Vulgaris* Powder (Ecofactory Co., Ltd., Seoul, South Korea). The environmental conditions for rotifers culture were maintained as water temperature 25.5 ± 0.1 °C, dissolved oxygen (DO) 6.6 ± 0.6 mg/L, salinity 25.0 ± 0.2 ppt and pH 7.5 ± 0.5. Rotifers were starved for 8 h prior to enrichment to ensure that any *Chlorella* previously ingested was cleared from their guts. The rotifers were then removed from the culture tanks, rinsed with seawater and transferred to small culture containers (2 L) with aeration. During the enrichment period, water quality parameters were maintained as per the previous culture conditions. Enrichment was carried out for 2 h with the addition of materials for each experimental group. Five experimental rotifer groups consisted of non-enriched group (RN) and enriched groups with 0.5% SCH (RS), RS + 0.1% taurine (RS+T), RS + 0.1% IMP (RS+I) and 0.5% *Chlorella* powder (RCL). The enriched rotifers were transferred to a 55 µm mesh filter and carefully washed with seawater. The rotifers were examined under an optical microscope (DM750, Leica Microsystems) fitted with a digital camera (ICC50E, Leica Microsystems, Heerbrugg, Switzerland) to verify the enrichment (Fig 1). After shrimp feeding, the remaining rotifers were stored at -82 °C for the analysis of proximate composition and fatty acid profiles.

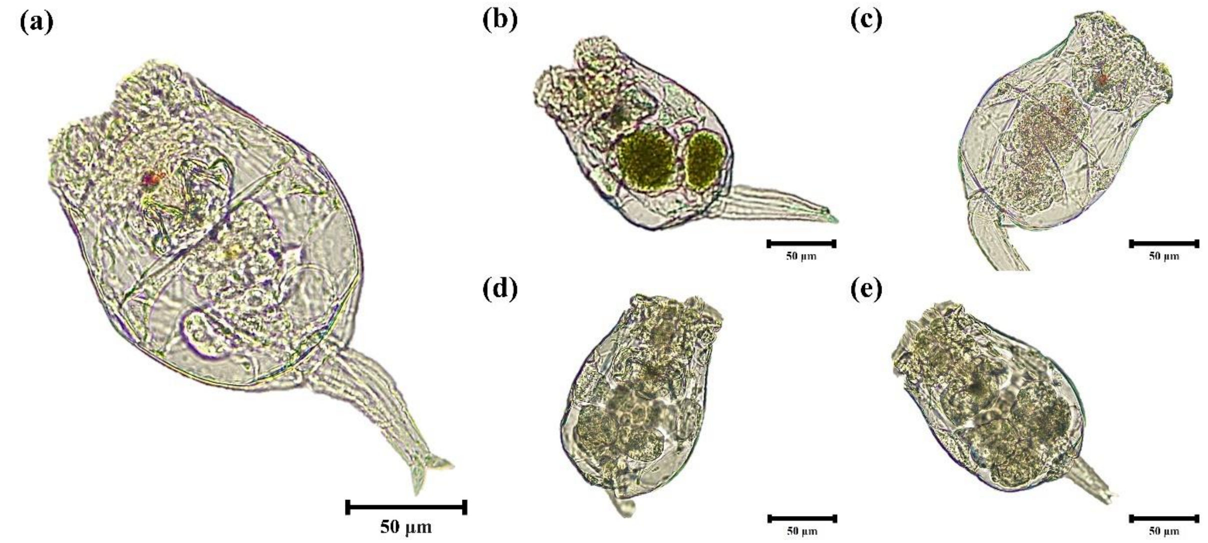

Fig 1. Microscopic visualization of the rotifers *Brachionus rotundiformis* after 2 h enrichment with each treatment. (a) non-enriched rotifers, (b) enriched rotifers by chlorella powder, (c) enriched rotifers by *Schizochytrium*, (d) enriched rotifers by *Schizochytrium* with 0.1% taurine, (e) enriched rotifers by *Schizochytrium* with 0.1% inosine monophosphate. Scale bar: 50 µm; Original magnification × 100.

Artemia culture and enrichment

Artemia cysts (Sep-art *Artemia*, INVE, Salt Lake City, UT, USA) were hatched using a commercial incubator (2 L), culture and enriched [19]. The hatching conditions were density 1.0 g/L, water temperature 30.1 ± 0.1 °C, salinity 30.5 ± 0.1 ppt and pH 7.0 ± 0.5 for 24 h. After hatching, a magnetic bar was used to remove the shells. *Artemia* were allowed to consume the yolk for 22 h. Enrichment was then performed for 2 h under each enrichment condition. F[illegible] experimental *Artemia* groups consisted of non-enriched group (AN) and enriched groups with 0.5% SCH (AS), AS + 0.1% taurine (AS+T), AS + 0.1% IMP (AS+I) and 0.5% *Chlorella* powder (ACL). The enriched *Artemia* were separated using a 0.1 mm sieve and then rinsed with freshwater to remove debris. The *Artemia* were examined under an optical microscope (DM750, Leica Microsystems) fitted with a digital camera (ICC50E, Leica Microsystems, Heerbrugg, Switzerland) to verify the enrichment (Fig 2). The remaining *Artemia* after feeding to the shrimp were stored at -82 °C for analysis of proximate composition and fatty acid.

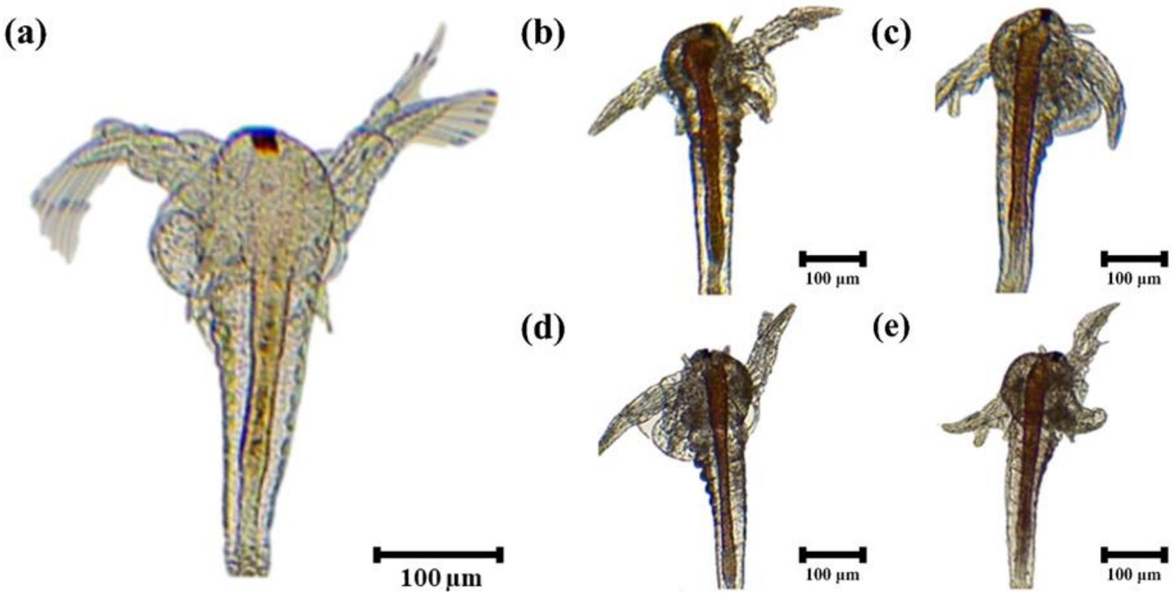

Fig 2. Microscopic visualization of the *Artemia* after 2 h enrichment with each treatment. (a) non-enriched *Artemia*, (b) enriched *Artemia* by chlorella powder, (c) enriched *Artemia* by *Schizochytrium*, (d) enriched *Artemia* by *Schizochytrium* with 0.1% taurine, (e) enriched *Artemia* by *Schizochytrium* with 0.1% inosine monophosphate. Scale bar: 100 µm; Original magnification × 40.

Feeding trial

Pacific white shrimp were obtained from a shrimp hatchery (Muan, South Korea). In the EXP-1, a total of 4,000 shrimp of PL1-2 stage were distributed at a density of 200 shrimp per tank (96 L), with four replicates per experimental group. The initial body length of the shrimp was 0.67 ± 0.03 mm. Aeration was set up for each tank to provide DO and the photoperiod was maintained at 12 h dark:12 h light using fluorescent lamps. During the EXP-1, water quality parameters were maintained as DO 6.43 ± 0.30 mg/L, water temperature 30.01 ± 0.98 ℃, pH 7.2 ± 0.7 and ammonia 0.06 ± 0.01 mg/L. At the start of the EXP-1, the rotifer feeding amount was 80 rotifers per shrimp, which was increased by 20% daily. The shrimp were fed the experimental rotifers three times a day (8:30, 14:30 and 20:30 h) for 9 days.

In the EXP-2, a total of 2,000 shrimp of PL12-14 stage were distributed at a density of 100 shrimp per tank (96 L), with four replicates per experimental group. The initial body weight of the shrimp was 3.67 ± 0.23 mg. The experimental environment was set up similarly to EXP-1. During the EXP-2, water quality parameters were maintained as DO 6.87 ± 0.21 mg/L, water temperature 29.81 ± 0.89 °C, pH 7.0 ± 0.4 and ammonia 0.08 ± 0.02 mg/L. At the start of the EXP-2, the *Artemia* feeding amount was 80 *Artemia* per shrimp, which was increased by 10% daily. The shrimp were fed the experimental *Artemia* three times a day (8:30, 14:30 and 20:30 h) for 12 days.

Proximate composition and fatty acid profile

The proximate composition of SCH, rotifers and *Artemia* was analyzed based on the AOAC [20] method. Moisture was measured by the amount remaining after drying at 125 °C for 3 h. Crude ash was measured by the amount remaining after roasting at 550 °C for 4 h. Crude protein was determined using a Kjeldahl nitrogen digester (SpeedDigester K-439, Buchi, Gwangmyeong, Korea). Crude lipid was analyzed based on the Folch et al. [21] method. The fatty acid profile of enrichment sources, enriched rotifers, enriched *Artemia* and whole-body of shrimp was analyzed by the gas chromatography method of Garcés and Mancha [22] using an 800GC (Agilent, CA, USA).

Growth performance and survival

At the end of the feeding trials, all shrimp were individually measured for length, weight and number. In EXP-1, length gain (LG) was calculated based on the final body length (FBL) of the shrimp. In EXP-2, weight gain (WG) was calculated based on the final body weight (FBW) of the shrimp. Survival was calculated based on the final number of shrimp in both experiments.

Hepatopancreas sample collection

Fifteen shrimp per tank (60 shrimp per experimental group) were anesthetized in ice water. Subsequently, the hepatopancreas was collected and rapidly froze using liquid nitrogen. The hepatopancreas samples were stored at -80 °C until the analysis of gene expression.

Quantitative real-time polymerase chain reaction (qRT-PCR)

To measure relative gene expression, RNA was extracted from the shrimp hepatopancreas using TRIzol® (Sigma, MO, USA) following the manufacturer's instructions. The OD_{260}/OD_{280} of the RNA extracts was measured using a Nano Drop 2000 (Thermo Scientific, NC, USA) to confirm the purity, which was between 1.8-2.0, indicating RNA extract. Afterwards, cDNA was synthesized using PrimeScript™ first-strand cDNA synthesis kit (Takara Bio, Shiga, Japan). PCR was conducted using the Real Time System TP 950 Thermal Cycler DiceTM (TP950, TaKaRa Bio, Shiga, Japan), with a reaction mixture containing 2 µL of cDNA, 10 µL of TaKaRa Ex TaqTM SYBR premix, 0.4 µL of forward and reverse primers, respectively and 7.2 µL of H_2O to make a total volume of 20 µL. The qRT-PCR analysis was performed using SYBR Premix Ex TaqTM Perfect Real-Time Kits (RR820A, TaKaRa Bio, Shiga, Japan) with 1 cycle of 95 °C for 30 s and 40 cycles of 94 °C for 15 s, 58 °C for 20 s and 72 °C for 20 s. Relative expression levels of each gene were calculated based on the Pfaffl [23] method. *β-actin* was used as the housekeeping gene. qPCR efficiency (E, factor) was measured using a five-fold serial dilution of pooled cDNA, and efficiency (%) was calculated from the slope of the standard curve. Reference gene stability (M value) was evaluated using geNorm software [24] based on Ct values across all samples. β-actin showed an M value of 0.5, indicating suitable stability under the experimental conditions. The sequences of the primers used in this study are presented in Table 2.

Table 2. Primers used for quantitative real-time quantitative PCR.

Gene	Sequence (5′-3′)	Accession number	Efficiency (%)
Lysozyme	F-TGTTCCGATCTGATGTCC<br>R-GCTGTTGTAAGCCACCC	AY170126.2	97.5

Crustin	F-ACGAGGCAACCATGAAGG<br>R-AACCACCACCAACACCTAC	AF430076	99.0
LGBP	F-CAGGGGCAACGACAACTTTG<br>R-GTGTGGGGATCTACTGCTCG	EU102286.1	96.3
Pen-3a	F-CACCCTTCGTGAGACCTTTG<br>R-AATATCCCTTTCCCACGTGAC	Y14926.1	98.2
proPO	F-CGGTGACAAAGTTCCTCTTC<br>R-GCAGGTCGCCGTAGTAAG	AY723296.1	97.8
FABP	F: CGCTAAGCCCGTGCTGGAAGT<br>R: CTCCTCGCCGAGCTTGATGGT	DQ398572.1	95.7
FAS	F: CAGGTGGAGATGCTCCTCGTGTT<br>R: GGTGACTAGCTCGGCTACATGGTT	HM595630.1	96.9
FATP	F: GACGGGCAAAGCGACTGA<br>R: ATGGACAAAGCCACGGAG	XM_027366808.1	98.5
CPT1	F: ACTCCCGATAAGCACACC<br>R: TTCATACATCCACCCCCT	XM_027373671.1	97.2
β-actin	F: CCACGAGACCACCTACAAC<br>R: AGCGAGGGCAGTGATTTC	AF300705.2	100.2

Abbreviations: *LGBP*, lipopolysaccharide-β-glucan binding protein; *Pen-3a*, penaeidin-3a; *proPO*, prophenoloxidase; *FABP*, fatty acid-binding protein; *FAS*, fatty acid synthase; *FATP*, fatty acid transport protein; *CPT1*, carnitine palmitoyl-transferase 1.

Statistical analysis

For statistical analysis, percentage data were transformed using the arcsine square root method. Data were analyzed using a one-way analysis of variance (ANOVA). When significant differences among dietary groups were identified by ANOVA, Tukey's honestly significant difference post-hoc test was conducted for multiple comparisons between groups. Results are expressed as mean ± standard deviation. All statistical analyses were performed using SPSS software (IBM SPSS Statistics 24, Corp., Armonk, NY, USA), with $P < 0.05$ considered statistically significant.

Results

Proximate composition and fatty acid profile of enriched rotifers and *Artemia*

In EXP-1, whole-body protein and crude lipid levels were significantly higher in all SCH-enriched rotifers than in RN and RCL rotifers (Table 3). Ash and moisture levels in all the rotifers did not show significant differences. For whole-body fatty acids, myristic acid (C14) was not detected in RCL-enriched rotifers. Palmitic acid (C16) level was significantly higher in all SCH-enriched rotifers compared to RN and RCL rotifers. Heptadecenoic acid (C17:1) level was only detected (1.98%) in RCL-enriched rotifers. Stearic acid (C18) and oleic acid (C18:1n9c) levels were significantly higher in RN rotifers compared to all other enriched rotifers. Linoleic acid (C18:2n6c) and C18:3n3 levels were significantly higher in RCL-enriched rotifers compared to all other enriched rotifers. C22:6n3 level was significantly higher in all the SCH-enriched rotifers compared to RN and RCL-enriched rotifers. In EXP-2, whole-body protein and crude lipid levels were significantly higher in all enriched *Artemia* than in AN *Artemia* (Table 4). Ash and moisture levels in all enriched *Artemia* did not show significant differences. Fatty acid analysis of the enriched *Artemia* showed that the levels of C16, palmitoleic acid (C16:1), C18, C181n9c, C18:2n6c, C18:3n3, C20:5n3 and C22:6n3 were significantly higher in all SCH-enriched *Artemia* compared to AN and ACL *Artemia*.

Table 3. Proximate composition and fatty acid profile of enriched rotifers.

	Rotifers				
	RN	RCL	RS	RS+T	RS+I
Proximate composition (%, wet basis)					
Crude protein	5.05±0.14^{b}	5.76±0.27^{b}	7.21±0.59^{a}	7.75±0.35^{a}	7.88±0.24^{a}
Crude lipid	9.35±0.31^{b}	10.5±0.36^{b}	13.4±0.57^{a}	13.4±0.15^{a}	13.9±0.12^{a}
Crude ash	0.86±0.05	0.87±0.04	0.82±0.05	0.84±0.05	0.86±0.04
Moisture	82.6±0.21	82.5±0.60	81.1±0.61	81.0±0.92	81.1±0.11
Fatty acid (%, rotifer)					
C14	0.32±0.00^{a}	0.00±0.00^{b}	0.30±0.01^{a}	0.30±0.02^{a}	0.33±0.01^{a}
C16	3.26±0.11^{b}	2.08±0.02^{c}	6.80±0.10^{a}	6.85±0.08^{a}	7.14±0.14^{a}
C17:1	0.00±0.00^{b}	1.98±0.01^{a}	0.00±0.00^{b}	0.00±0.00^{b}	0.00±0.00^{b}

C18	1.27±0.07[a]	0.00±0.00[c]	0.39±0.04[b]	0.36±0.00[b]	0.37±0.02[b]
C18:1n9c	2.37±0.09[a]	0.61±0.00[b]	0.00±0.00[c]	0.00±0.00[c]	0.00±0.00[c]
C18:2n6c	2.12±0.09[b]	5.40±0.00[a]	0.21±0.02[c]	0.25±0.03[c]	0.22±0.00[c]
C18:3n3	0.00±0.00[b]	0.44±0.00[a]	0.00±0.00[b]	0.00±0.00[b]	0.00±0.00[b]
C20:5n3	ND	ND	ND	ND	ND
C22:6n3	0.00±0.00[b]	0.00±0.00[b]	5.65±0.10[a]	5.65±0.12[a]	5.86±0.11[a]
∑SFA[1]	4.86±0.18[c]	2.08±0.02[b]	7.50±0.08[a]	7.51±0.09[a]	7.85±0.11[a]
∑MUFA[2]	2.37±0.09[b]	2.59±0.01[a]	0.00±0.00[c]	0.00±0.00[c]	0.00±0.00[c]
∑PUFA[3]	2.12±0.09[b]	5.84±0.01[a]	5.86±0.08[a]	5.90±0.09[a]	6.08±0.11[a]

The experimental groups consisted of non-enriched group (RN) and enriched groups with 0.5% chlorella powder (RCL), 0.5% SCH (RS), RS + 0.1% taurine (RS+T) and RS + 0.1% IMP (RS+I). Values are mean of quadruplicate (n=4) and presented as mean ± S.D. Values with different superscripts in the same row are significantly different ($P < 0.05$). ND, not detected.

[1]The sum saturated fatty acids

[2]The sum monounsaturated fatty acids

[3]The sum polyunsaturated fatty acids

Table 4. Proximate composition and fatty acid profile of enriched *Artemia*.

	Artemia				
	AN	ACL	AS	AS+T	AS+I
Proximate composition (%, wet basis)					
Crude protein	5.86±0.02[b]	6.06±0.04[a]	6.04±0.04[a]	6.16±0.00[a]	6.07±0.02[a]
Crude lipid	2.80±0.00[c]	4.39±0.18[b]	7.18±0.24[a]	6.96±0.24[a]	7.09±0.12[a]
Crude ash	0.93±0.06	0.90±0.06	0.92±0.03	0.93±0.03	0.99±0.09
Moisture	86.1±0.52	86.5±0.69	86.9±1.10	86.8±0.54	86.4±0.55
Fatty acid (%, Artemia)					
C16	0.41±0.02[c]	0.64±0.00[b]	1.40±0.00[a]	1.31±0.04[a]	1.39±0.05[a]
C16:1	0.13±0.00[c]	0.19±0.02[b]	0.27±0.01[a]	0.28±0.00[a]	0.29±0.00[a]
C18	0.32±0.00[c]	0.48±0.00[b]	0.67±0.01[a]	0.68±0.01[a]	0.68±0.01[a]
C18:1n9c	0.70±0.00[c]	1.08±0.03[b]	1.42±0.04[a]	1.40±0.02[a]	1.39±0.00[a]
C18:2n6c	0.21±0.00[c]	0.40±0.01[b]	0.46±0.01[a]	0.45±0.00[a]	0.45±0.00[a]
C18:3n3	0.94±0.00[d]	1.46±0.02[c]	1.93±0.01[a]	1.86±0.01[b]	1.89±0.02[ab]

C20:5n3	0.09±0.01^{b}	0.12±0.01^{b}	0.26±0.03^{a}	0.25±0.00^{a}	0.25±0.00^{a}
C22:6n3	0.01±0.00^{b}	0.02±0.00^{b}	0.78±0.01^{a}	0.73±0.01^{a}	0.74±0.05^{a}
∑SFA[1]	0.73±0.01^{c}	1.12±0.00^{b}	2.07±0.01^{a}	2.00±0.03^{a}	2.07±0.04^{a}
∑MUFA[2]	0.83±0.00^{c}	1.27±0.01^{b}	1.69±0.03^{a}	1.68±0.01^{a}	1.68±0.00^{a}
∑PUFA[3]	1.24±0.01^{d}	2.00±0.02^{c}	3.43±0.04^{a}	3.28±0.01^{b}	3.34±0.04^{ab}

The experimental groups consisted of non-enriched group (AN) and enriched groups with 0.5% chlorella powder (ACL), 0.5% SCH (AS), AS + 0.1% taurine (AS+T) and AS + 0.1% IMP (AS+I). Values are mean of quadruplicate (n=4) and presented as mean ± S.D. Values with different superscripts in the same row are significantly different ($P < 0.05$).

[1]The sum saturated fatty acids

[2]The sum monounsaturated fatty acids

[3]The sum polyunsaturated fatty acids

Growth performance and survival

In EXP-1, FBL and LG of the shrimp were significantly increased in all SCH-enrichment groups compared to RN and RCL groups (Table 5). Survival was significantly higher in RS+T group than in RN and RCL groups. In EXP-2, FBW and WG of shrimp were significantly increased in all SCH-enrichment groups compared to in AN and ACL groups (Table 6). Survival was not significantly different among all experimental groups.

Table 5. Growth performance and survival of Pacific white shrimp *Penaeus vannamei* (initial body length: 0.67 ± 0.03 mm) fed enriched rotifer for 9 days.

	Experimental groups				
	RN	RCL	RS	RS+T	RS+I
Final body length[1]	7.37±0.57^{b}	8.02±0.17^{b}	10.1±0.11^{a}	10.2±0.15^{a}	10.2±0.36^{a}
Length gain[2]	995±84^{b}	1091±25^{b}	1406±16^{a}	1413±23^{a}	1408±53^{a}
Survival (%)	43.5±11.7^{c}	55.1±9.3^{bc}	70.1±5.0^{ab}	73.9±6.5^{a}	68.5±3.3^{ab}

The experimental groups consisted of non-enriched group (RN) and enriched groups with 0.5% chlorella powder (RCL), 0.5% SCH (RS), RS + 0.1% taurine (RS+T) and RS + 0.1% IMP (RS+I). Values are mean of quadruplicate (n=4) and presented as mean ± S.D. Values with different superscripts in the same row are significantly different ($P < 0.05$).

[1]Final body length (mm)

[2]Length gain (%) = (Final body length - initial body length) / (initial body length) × 100

Table 6. Growth performance and survival of Pacific white shrimp *Penaeus vannamei* (initial body weight: 3.67 ± 0.23 mg) fed enriched *Artemia* for 12 days.

	Experimental groups				
	AN	ACL	AS	AS+T	AS+I
Final body weight[1]	19.5±0.2^{b}	19.6±1.2^{b}	22.6±0.5^{a}	22.7±0.6^{a}	24.2±0.8^{a}
Weight gain[2]	430±6^{b}	434±33^{b}	497±42^{a}	518±16^{a}	559±23^{a}
Survival (%)	94.8±3.1	94.0±5.2	97.0±1.0	97.8±1.1	97.5±2.7

The experimental groups consisted of non-enriched group (AN) and enriched groups with 0.5% chlorella powder (ACL), 0.5% SCH (AS), AS + 0.1% taurine (AS+T) and AS + 0.1% IMP (AS+I).
Values are mean of quadruplicate (n=4) and presented as mean ± S.D.
Values with different superscripts in the same row are significantly different (P < 0.05).
[1]Final body weight (mg)
[2]Weight gain (%) = [(final body weight - initial body weight) / initial body weight] × 100

Fatty acid profile of whole-body of shrimp

In EXP-1, fatty acid analysis in the whole body of shrimp fed rotifers enriched with each nutrient-enriched source for 9 days showed that all SCH enriched groups had significantly higher C22:6n3 level than did RN and RCL groups (Table 7). C18:3n3 level was significantly higher in RCL group compared to other groups. C16 level was significantly higher in RN compared to RS+T and RS+I groups. C18 level was significantly higher in RN compared to all the other groups. There were no significant differences in the C14, C16:1, C18:1n9c, C18:2n6c and C20:5n3 levels among all experimental groups. In EXP-2, fatty acid analysis of the whole body of shrimp fed with enriched *Artemia* as each nutrient-enriched source for 12 days showed that all SCH enriched groups had significantly higher C22:6n3 levels than AN and ACL groups (Table 8). C16 levels were significantly higher in AN and ACL groups compared to all SCH enriched groups. C18 level was significantly higher in AN and ACL groups compared to AS group. C18:1n9c level was significantly higher in AN

group compared to all other experimental groups. C18:3n3 level was significantly higher in ACL group compared to all other experimental groups. There were no significant differences in the C14, C16:1, C18:2n6c and C20:5n3 levels among all experimental groups.

Table 7. Fatty acid profile in whole body of Pacific white shrimp *Penaeus vannamei* fed the experimental rotifers for 9 days.

Fatty acid (%, lipids)	Experimental groups				
	RN	RCL	RS	RS+T	RS+I
C14	4.66±0.00	4.53±0.28	4.41±0.02	4.49±0.18	4.34±0.17
C16	29.5±0.40^{a}	21.8±1.96^{ab}	21.6±2.09^{ab}	20.5±2.91^{b}	21.4±0.88^{b}
C16:1	4.26±0.15	4.12±0.13	4.08±0.07	4.19±0.24	4.12±0.14
C18	26.3±0.01^{a}	19.1±0.22^{b}	19.1±0.29^{b}	19.4±0.52^{b}	19.3±0.07^{b}
C18:1n9c	4.78±0.25	4.43±0.15	4.32±0.00	4.41±0.56	4.22±0.15
C18:2n6c	7.49±0.24	7.55±0.43	7.56±0.34	7.39±0.25	7.46±0.12
C18:3n3	5.68±0.30^{b}	18.3±0.04^{a}	5.69±0.28^{b}	5.55±0.10^{b}	5.59±0.53^{b}
C20:5n3	8.56±0.10	11.4±1.48	11.1±0.29	11.1±0.40	11.1±0.75
C22:6n3	8.76±0.29^{b}	8.80±0.23^{b}	22.2±1.50^{a}	23.0±0.64^{a}	22.5±0.53^{a}
∑SFA[1]	60.5±0.40^{a}	45.4±2.46^{b}	45.1±1.77^{b}	44.3±2.20^{b}	45.0±0.64^{b}
∑MUFA[2]	9.04±0.10	8.55±0.28	8.40±0.06	8.60±0.81	8.34±0.02
∑PUFA[3]	30.5±0.32^{b}	46.1±2.18^{a}	46.6±1.84^{a}	47.1±1.39^{a}	46.6±0.63^{a}

The experimental groups consisted of non-enriched group (RN) and enriched groups with 0.5% chlorella powder (RCL), 0.5% SCH (RS), RS + 0.1% taurine (RS+T) and RS + 0.1% IMP (RS+I). Values are mean of quadruplicate (n=4) and presented as mean ± S.D. Values with different superscripts in the same row are significantly different ($P < 0.05$).

[1]The sum saturated fatty acids

[2]The sum monounsaturated fatty acids

[3]The sum polyunsaturated fatty acids

Table 8. Fatty acid profile in whole body of Pacific white shrimp *Penaeus vannamei* fed the experimental *Artemia* for 12 days.

Fatty acid	Experimental groups

(%, lipids)	AN	ACL	AS	AS+T	AS+I
C14	4.33±0.17	4.23±0.03	4.29±0.10	4.31±0.03	4.22±0.00
C16	21.3±0.02^{a}	20.9±0.33^{a}	18.4±0.07^{b}	18.4±0.15^{b}	18.5±0.38^{b}
C16:1	4.22±0.01	4.27±0.02	4.34±0.14	4.20±0.10	4.25±0.02
C18	18.9±0.07^{a}	19.0±0.02^{a}	14.7±0.02^{b}	15.5±0.22^{ab}	16.3±2.17^{ab}
C18:1n9c	16.1±0.64^{a}	12.7±0.77^{b}	13.9±0.10^{b}	13.4±0.10^{b}	13.2±0.28^{b}
C18:2n6c	5.88±1.21	4.91±0.31	5.39±0.03	5.25±0.06	5.26±0.42
C18:3n3	13.4±0.18^{b}	18.4±0.32^{a}	13.4±0.01^{b}	13.3±0.08^{b}	13.5±0.29^{b}
C20:5n3	11.1±0.02	11.4±0.39	12.0±0.01	11.7±0.08	11.6±0.70
C22:6n3	4.86±0.17^{b}	4.24±0.77^{b}	13.6±0.21^{a}	13.9±0.02^{a}	13.3±1.47^{a}
∑SFA[1]	44.5±0.25^{a}	44.1±0.34^{a}	37.4±0.20^{b}	38.3±0.04^{b}	39.0±1.79^{b}
∑MUFA[2]	20.3±0.63^{a}	16.9±0.75^{b}	18.2±0.04^{b}	17.6±0.00^{b}	17.4±0.26^{b}
∑PUFA[3]	35.1±0.88^{b}	39.0±0.41^{b}	44.4±0.16^{a}	44.2±0.04^{a}	43.6±2.04^{a}

The experimental groups consisted of non-enriched group (AN) and enriched groups with 0.5% chlorella powder (ACL), 0.5% SCH (AS), AS + 0.1% taurine (AS+T) and AS + 0.1% IMP (AS+I). Values are mean of quadruplicate (n=4) and presented as mean ± S.D. Values with different superscripts in the same row are significantly different ($P < 0.05$).

[1]The sum saturated fatty acids

[2]The sum monounsaturated fatty acids

[3]The sum polyunsaturated fatty acids

Relative gene expression related to lipid metabolism

In EXP-1, the shrimp hepatopancreas in all SCH-enriched groups showed significantly upregulated gene expressions of fatty acid-binding protein (*FABP*), fatty acid synthase (*FAS*), fatty acid transport protein (*FATP*) and carnitine palmitoyl-transferase 1 (*CPT1*) compared to RN and RCL groups (Fig 3). In addition, *FAS* gene expression was significantly upregulated in the RCL group compared to RN group. In EXP-2, the shrimp hepatopancreas in all the SCH-enriched groups showed significantly upregulated gene expressions of *FABP*, *FAS*, *FATP* and *CPT1* compared to AN group (Fig 4).

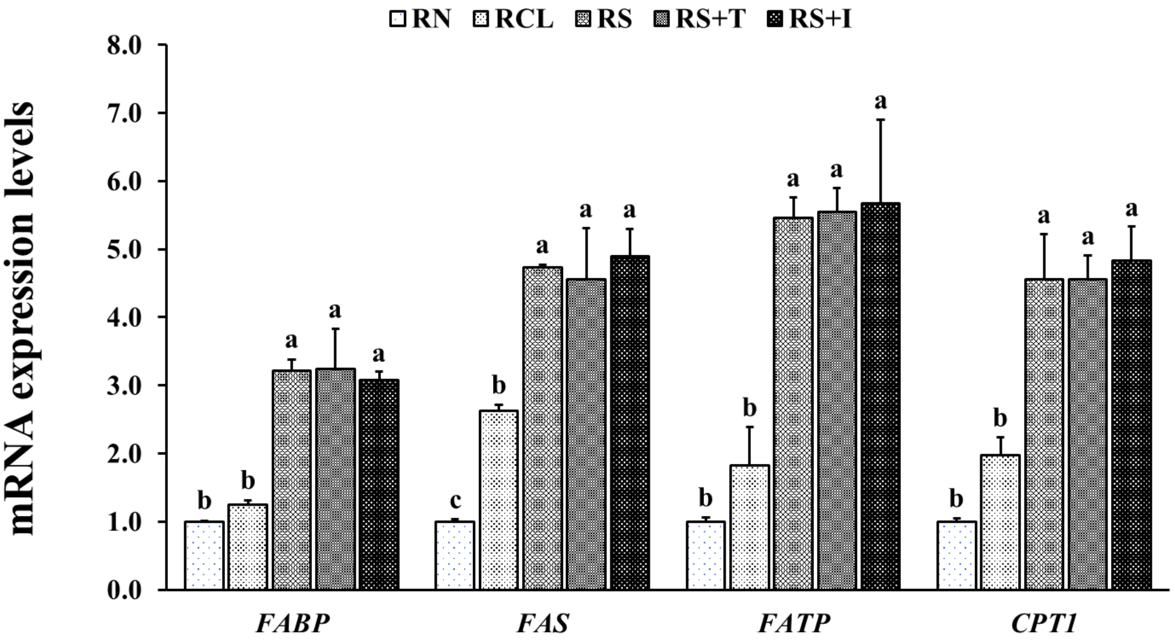

Fig 3. Relative mRNA expressions of fatty acid-binding protein (*FABP*), fatty acid synthase (*FAS*), fatty acid transport protein (*FATP*) and carnitine palmitoyl-transferase 1 (*CPT1*) in the hepatopancreas of Pacific white shrimp *Penaeus vannamei* fed rotifers enriched with each treatment for 9 days. Bars with different letters are significantly different ($P < 0.05$).

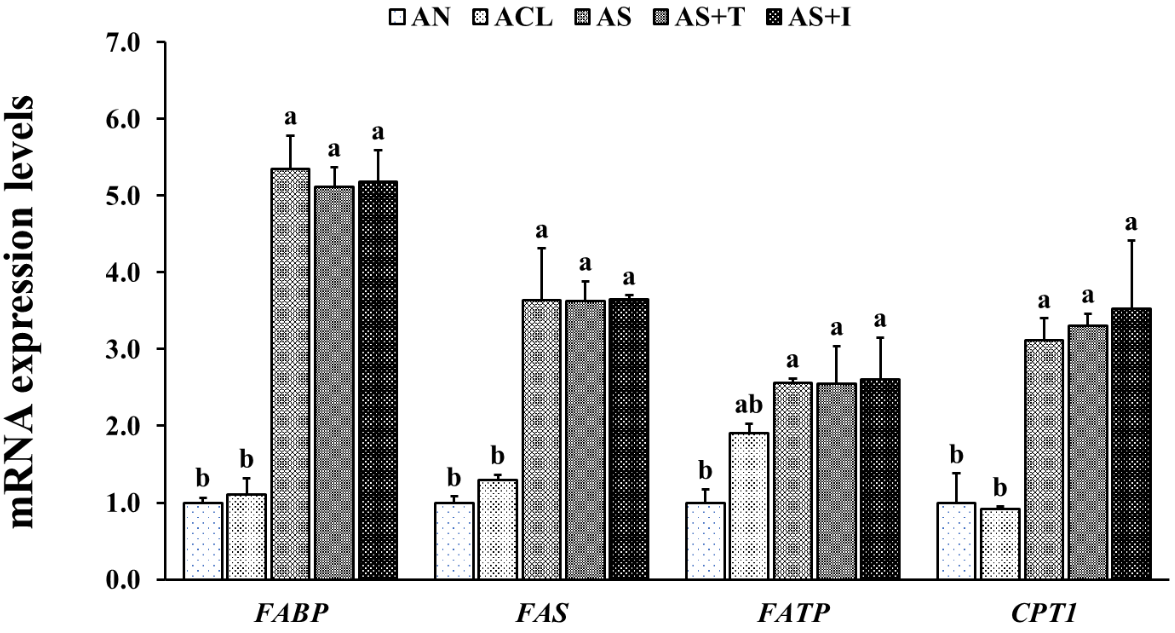

Fig 4. Relative mRNA expressions of fatty acid-binding protein (*FABP*), fatty acid synthase (*FAS*), fatty acid transport protein (*FATP*) and carnitine palmitoyl-transferase 1 (*CPT1*) in the

hepatopancreas of Pacific white shrimp *Penaeus vannamei* fed *Artemia* nauplii enriched with each treatment for 12 days. Bars with different letters are significantly different (P < 0.05).

Relative gene expression related to innate immunity

In EXP-1, *lysozyme* expression in the hepatopancreas of shrimp was significantly upregulated in all the enrichment groups compared to the RN group (Fig 5). *Crustin*, lipopolysaccharide-β-glucan binding protein (*LGBP*) and prophenoloxidase (*proPO*) expression were significantly upregulated in all the SCH enrichment groups compared to RN and RCL groups, with RS+I group showing the highest expression. Penaeidin-3a (*Pen-3a*) expression was significantly upregulated in all the SCH enrichment groups compared to RN and RCL groups. In EXP-2, *lysozyme* expression in the hepatopancreas of shrimp was significantly upregulated in AS+T group compared to AN and ACL groups (Fig 6). *Crustin* expression was significantly upregulated in all the SCH enrichment groups compared to AN and ACL groups, with AS+I showed the highest expression. *LGBP* expression was significantly upregulated in all the SCH enrichment groups compared to AN and ACL groups, with AS+T group which showed the highest expression. *Pen-3a* and *proPO* expression were significantly upregulated in the AS, AS+T and AS+I groups compared to AN group.

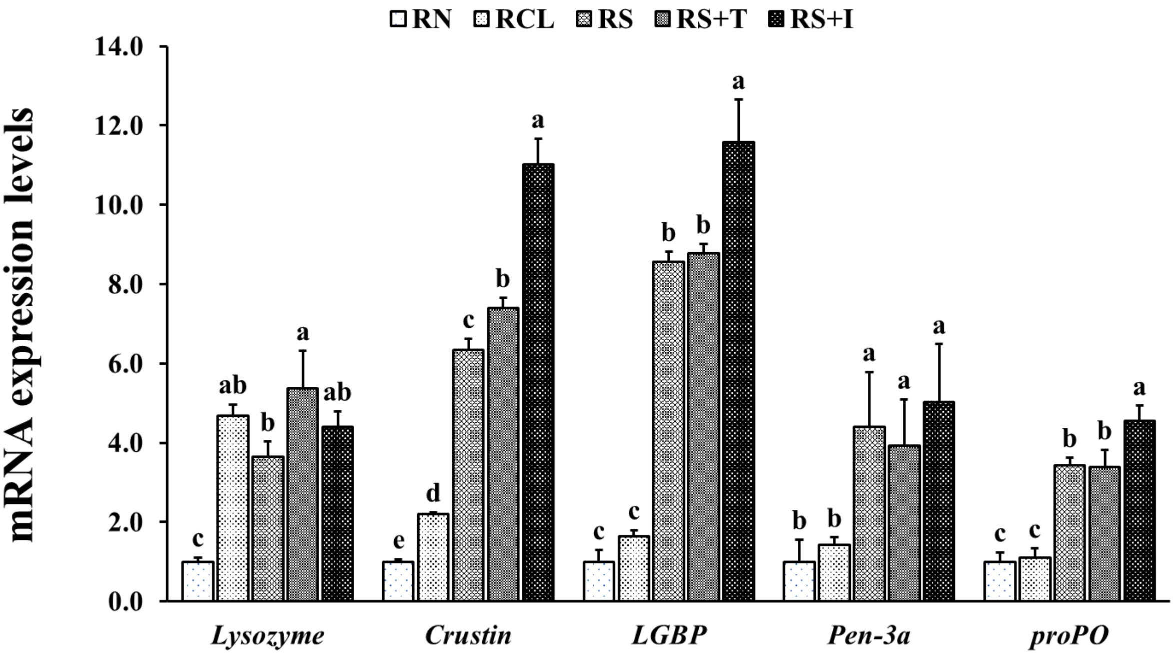

Fig 5. Relative mRNA expressions of *lysozyme*, *crustin*, lipopolysaccharide-β-glucan binding protein (*LGBP*), penaeidin-3a (*Pen-3a*) and prophenoloxidase (*proPO*) in the hepatopancreas of Pacific white shrimp *Penaeus vannamei* fed rotifers enriched with each treatment for 9 days. Bars with different letters are significantly different ($P < 0.05$).

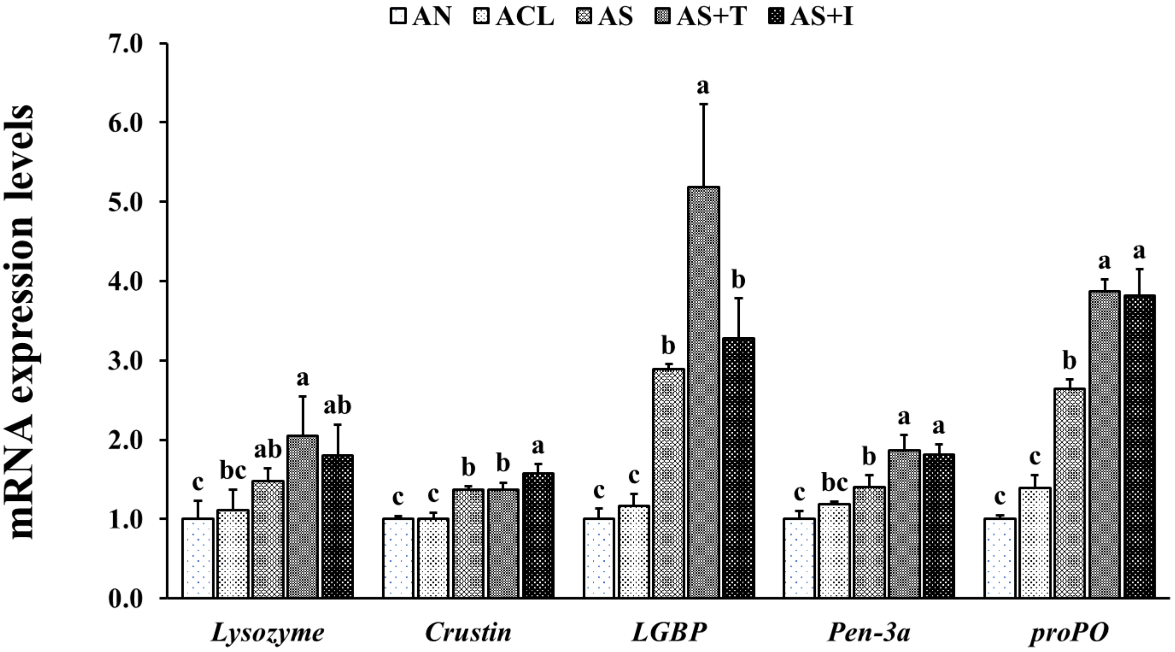

Fig 6. Relative mRNA expressions of *lysozyme*, *crustin*, lipopolysaccharide-β-glucan binding protein (*LGBP*), penaeidin-3a (*Pen-3a*) and prophenoloxidase (*proPO*) in the hepatopancreas of

Pacific white shrimp *Penaeus vannamei* fed *Artemia* nauplii enriched with each treatment for 12 days. Bars with different letters are significantly different (*P* < 0.05).

Discussion

In the present study, the significant increase in DHA level in rotifers and *Artemia* enriched with SCH is consistent with previous studies showing that these live feeds can serve as carriers of essential fatty acids such as DHA [5]. Similarly, previous studies on silver pompano and turbot have shown that feeding SCH-enriched rotifers or *Artemia* enhances whole-body DHA content and improves both growth and survival [9, 10]. SCH enrichment significantly improved growth in both feeding trials, with higher survival observed in EXP-1 compared to the non-enriched and *Chlorella*-enriched groups. These improvements in growth and survival are likely attributable to the elevated dietary DHA levels resulting from SCH enrichment. Numerous studies have reported that increasing dietary DHA levels enhances the growth and survival of Pacific white shrimp [13, 14, 25]. Specifically, when the white shrimp are cultured under high salinity conditions (30 ppt), their ability to synthesize polyunsaturated fatty acids (PUFA) such as EPA and DHA, can be limited, making dietary inclusion of PUFA essential [14]. PUFA are required at high levels during larval stages due to their proportionately higher requirements in the development of visual and neural tissues as precursors for cell membrane structure, energy metabolism and hormone synthesis [26]. Suprayudi et al. [27] emphasized that a deficiency in dietary PUFA can lead to molting failure in crustacean larvae, which can significantly impact growth and survival. In this study, we propose that dietary DHA supplementation through SCH enrichment of two species of zoo-live feed increases whole-body PUFA levels in shrimp, thereby improving their growth and survival.

The hepatopancreas is a vital organ responsible for lipid storage, synthesis, secretion of digestive enzymes and energy metabolism [28]. This study investigated the effects of dietary SCH on lipid metabolism in the hepatopancreas by examining the gene expression of *FAS*, *CPT1*, *FABP* and *FATP*.

Shrimp fed SCH-enriched rotifers and *Artemia* showed higher expression levels of all lipid metabolism-related genes compared to those fed non-enriched or *Chlorella*-enriched live feeds. This upregulation corresponded with the elevated levels of PUFAs, particularly DHA, as well as increased protein and total lipid contents in the enriched live feeds, which were subsequently transferred to the shrimp. Greater lipid availability provides more fatty acid substrates for synthesis and oxidation, while higher protein levels support the production of enzymes and regulatory proteins involved in lipid metabolism [29, 30]. Together, these nutritional improvements can activate lipid-sensitive transcription factors such as peroxisome proliferator-activated receptors and sterol regulatory element-binding proteins [26], thereby enhancing the transcription of genes associated with fatty acid synthesis, transport and oxidation. Additionally, DHA incorporation into membrane phospholipids influences membrane properties and the function of membrane-bound proteins, including those involved in lipid metabolism [31]. These mechanisms explain how dietary SCH modulates lipid metabolism and supports shrimp health. Specifically, *FAS* catalyzes fatty acid synthesis and maintains lipid homeostasis [32]; *CPT1* regulates mitochondrial fatty acid oxidation for energy production [33]; *FABP* facilitates intracellular transport of fatty acids [34]; and *FATP* mediates fatty acid uptake across cell membranes, contributing to lipid metabolism and energy balance [35]. In summary, our findings provide the first evidence that SCH-enriched live feeds enhance lipid metabolism in shrimp post-larvae by improving dietary PUFA supply and proximate composition, thereby promoting growth, development, survival and energy metabolism.

This study highlights the potential of SCH as an immune-enhancing supplement in the post-larvae culture of Pacific white shrimp. Enrichment of rotifers and *Artemia* with SCH upregulated the expression of key immune-related genes, including *lysozyme*, *crustin*, *LGBP*, *Pen-3a* and *proPO*. Given that invertebrates lack an adaptive immune system, the innate immune system is important in defending shrimp against pathogens [36]. *Lysozyme*, in particular, plays an important role in immune defense with its lytic activity against various cell wall pathogens, including *Vibrio* spp. [37]. *Crustin* and *Pen-3a* are antimicrobial peptides that provide protection against various pathogens, including

Gram-positive bacteria and fungi [38]. *LGBP* is a pattern recognition protein that, upon binding to pathogen ligands activates the *proPO* system, which is crucial for melanization [36]. The upregulation of these immune genes in the SCH-enriched group may be attributed to the increased levels of PUFA observed in the whole-body composition of the shrimp. PUFA are vital for immune regulation because they participate in numerous cellular functions, including signaling, maintaining cell membrane fluidity and structure, and regulating the nervous system [26]. Although the precise mechanisms by which PUFA enhance immunity in shrimp remain unclear, Chen et al. [39] reported that PUFA-mediated induction of immune gene expression could enhance antibacterial defense and improve survival against *Vibrio parahaemolyticus* infection. Another hypothesis for the enhancement of the shrimp immune system by SCH is that the polysaccharides present in SCH may stimulate the innate immune system. Chen et al. [40] demonstrated that shrimp hemocytes exposed to algal polysaccharides and extracts can trigger the recognition and binding of *LGBP*, induce degranulation and activate the *proPO* system, thereby promoting innate immunity. Furthermore, Chen et al. [41] found that Pacific white shrimp fed diets supplemented with Spirulina powder (30–60 g/kg) exhibited enhanced resistance to *V. alginolyticus* infection due to the activation of innate immune factors such as *lysozyme*, *LGBP* and phenoloxidase (PO). This study demonstrates that SCH can act as a potent immune stimulator in Pacific white shrimp larvae by enhancing the expression of key immune genes and activating innate immune responses. These findings suggest that incorporating SCH into live feeds may significantly boost immunity and improve survival in shrimp post-larvae.

Our study demonstrated that the addition of taurine and IMP to the enrichment process yielded greater immune-enhancing effects than SCH alone. Although research on the impact of taurine on innate immune gene expression in shrimp is limited, exogenous taurine has been reported to promote innate immune gene expression and enhance antibacterial activity in the Chinese mitten crab (*Eriocheir sinensis*) [42] and zebrafish (*Danio rerio*) [43]. Wang et al. [15] suggested that taurine metabolism is regulated in Pacific white shrimp infected with *Vibrio* spp., potentially enhancing antibacterial responses and survival. Additionally, dietary nucleotides, including IMP, are known to

enhance immune responses in aquatic animals [18]. While the mechanisms underlying the immunostimulatory effects of nucleotides in shrimp remain unclear, Shankar et al. [44] demonstrated that dietary nucleotides stimulate innate immune responses, such as PO activity and total hemocyte count, in freshwater prawns (*Macrobrachium rosenbergii*), thereby improving resistance against Aeromonas hydrophila–induced white muscle disease. Similarly, Xiong et al. [45] found that feeding nucleotide-enriched yeast to Pacific white shrimp enhanced serum PO and lysozyme activities. Our findings are consistent with previous studies indicating that taurine and nucleotides can significantly enhance immune responses in various aquatic species. This suggests a synergistic interaction where SCH improves membrane fatty acid and PUFA supply, while taurine and IMP modulate gene transcription, hemocyte proliferation and immune effector function. Although the positive effects of taurine and IMP supplementation on immunity have been demonstrated, further research is required to elucidate the mechanisms of action and to determine the optimal enrichment concentrations.

Conclusion

Our study demonstrates that SCH enrichment of zoo-live feeds results in higher DHA accumulation, which in turn improves growth, lipid metabolism, innate immune responses and survival in Pacific white shrimp post-larvae. Furthermore, supplementation with taurine and IMP shows potential for enhancing immune function. However, further research is needed to better understand the underlying mechanisms and establish the optimal enrichment levels.

Author contributions

Writing – Original draft: Suhyeok Kim

Formal analysis: Sanghyun Song, Yeonji Lee, Yein Lee

Writing – Review & Editing: Kyeong-Jun Lee

Funding acquisition: Young-Mi Lee, Kook-Jin Cho

Conceptualization: Kyeong-Jun Lee

Supervision: Kyeong-Jun Lee

Acknowledgment

This work was supported by the CJ Cheiljedang Co., Ltd and Basic Science Research Program through the National Research Foundation of Korea (NRF) funded by the Ministry of Education (RS-2019-NR040078)

References

1. FAO, The State of World Fisheries and Aquaculture. Towards Blue Transformation. Rome, FAO. 2022, https://doi.org/10.4060/cc0461en
2. Rojo-Arreola, L., García-Carreño, F., Romero, R., Díaz Dominguez, L., 2020. Proteolytic profile of larval developmental stages of *Penaeus vannamei*: An activity and mRNA expression approach. PLoS One. 15(9), e0239413. https://doi.org/10.1371/journal.pone.0239413
3. Jamali, H., Imani, A., Abdollahi, D., Roozbehfar, R., Isari, A., 2015. Use of probiotic Bacillus spp. in rotifer (*Brachionus plicatilis*) and Artemia (*Artemia urmiana*) enrichment: Effects on growth and survival of Pacific white shrimp, *Litopenaeus vannamei*, Larvae. Probiotics Antimicrob Proteins. 7, 118-125. https://doi.org/10.1007/s12602-015-9189-3
4. Bengtson, D.A., 2003. Status of marine aquaculture in relation to live prey: past, present and future. Live feeds in marine aquaculture. pp.1-16.
5. Dineshbabu, G., Goswami, G., Kumar, R., Sinha, A., Das, D., 2019. Microalgae–nutritious, sustainable aqua-and animal feed source. J Funct Foods. 62, 103545. https://doi.org/10.1016/j.jff.2019.103545
6. Kandathil Radhakrishnan, D., AkbarAli, I., Schmidt, B.V., John, E.M., Sivanpillai, S., Thazhakot

Vasunambesan, S., 2020. Improvement of nutritional quality of live feed for aquaculture: An overview. Aquac Res. 51(1), 1-17. https://doi.org/10.1111/are.14357

7. Kousoulaki, K., Østbye, T.K.K., Krasnov, A., Torgersen, J.S., Mørkøre, T., Sweetman, J., 2015. Metabolism, health and fillet nutritional quality in Atlantic salmon (*Salmo salar*) fed diets containing n-3-rich microalgae. J Nutr Sci. 4, e24. https://doi.org/10.1017/jns.2015.14

8. Kumar, V., Habte-Tsion, H.M., Allen, K.M., Bowman, B.A., Thompson, K.R., El-Haroun, E., Keith, F., Tidwell, J.H., 2018. Replacement of fish oil with *Schizochytrium* meal and its impacts on the growth and lipid metabolism of Pacific white shrimp (*Litopenaeus vannamei*). Aquac Nutr, 24(6). 1769-1781. https://doi.org/10.1111/anu.12816

9. Song, X., Zhang, X., Guo, N., Zhu, L., Kuang, C., 2007. Assessment of marine thraustochytrid *Schizochytrium limacinum* OUC88 for mariculture by enriched feeds. Fish Sci. 73, 565-573. https://doi.org/10.1111/j.1444-2906.2007.01369.x

10. Pedroso, F.L., Moquera, G.L., 2023. Utilization of thraustochytrid, *Schizochytrium* sp. as live food enrichment enhances growth performance, digestive enzyme activities, and stress tolerance of silver pompano (*Trachinotus blochii*) larvae. Int J Aquat Biol. 11(5), 439-448. https://doi.org/10.22034/ijab.v11i5.2017

11. Hemaiswarya, S., Raja, R., Ravi Kumar, R., Ganesan, V., Anbazhagan, C., 2011. Microalgae: a sustainable feed source for aquaculture. World J Microbiol Biotechnol. 27, 1737-1746. https://doi.org/10.1007/s11274-010-0632-z

12. Matsunari, H., Hashimoto, H., Oda, K., Masuda, Y., Imaizumi, H., Teruya, K., Furuita, H., Yamamoto, T., Hamada, K., Mushiake, K., 2013. Effects of docosahexaenoic acid on growth, survival and swim bladder inflation of larval amberjack (*Seriola dumerili*, Risso). Aquac Res. 44(11), 1696-1705. https://doi.org/10.1111/j.1365-2109.2012.03174.x

13. Feng, Z.F., Dong, W.X., Bu, F., Zhu, W., 2021. Influence of dietary n-3 polyunsaturated fatty acids (PUFAs) on growth, PUFA composition and the expression of FAD6 and ELOV5 in pacific white shrimp (*Litopenaeus vannamei*). Aquac Res. 52(4), 1666-1676. https://doi.org/10.1111/are.15019

14. Chen, K., Li, E., Gan, L., Wang, X., Xu, C., Lin, H., Qin J.G., Chen, L., 2014. Growth and lipid metabolism of the pacific white shrimp *Litopenaeus vannamei* at different salinities. J Shellfish Res. 33(3), 825-832. https://doi.org/10.2983/035.033.0317
15. Wang, Z., Aweya, J.J., Yao, D., Zheng, Z., Wang, C., Zhao, Y., Li, S., Zhang, Y., 2022. Taurine metabolism is modulated in *Vibrio*-infected *Penaeus vannamei* to shape shrimp antibacterial response and survival. Microbiome, 10(1), 213. https://doi.org/10.1186/s40168-022-01414-9
16. El-Sayed, A.F.M., 2014. Is dietary taurine supplementation beneficial for farmed fish and shrimp? A comprehensive review. Rev Aquac. 6(4), 241-255. https://doi.org/10.1111/raq.12042
17. Honikel, K.O., 2014. Encyclopedia of meat sciences, Conversion of muscle to meat (glycolysis), E-Publishing Inc., London, pp. 353 – 357.
18. Hossain, M.S., Koshio, S., Kestemont, P., 2020. Recent advances of nucleotide nutrition research in aquaculture: a review. Rev Aquac. 12(2), 1028-1053. https://doi.org/10.1111/raq.12370
19. Kim, S., Shin, J., Lim, H., Ko, D., Eom, G., Lim, J., Lee, Y., Choi, S., Park, S.Y., Park, J.Y., Lee, K.J., 2024. Effects of feeding poly-β-hydroxybutyrate enriched *Artemia* nauplii on the growth, digestibility and immunity of Pacific white shrimp (*Penaeus vannamei*) post-larvae. Fish Aquatic Sci. 27(7), 447-455. https://doi.org/10.47853/FAS.2024.e42
20. AOAC (Association of Official Analytical Chemists). Official methods of analysis of official analytical chemists international. 18th ed. Arlington, VA, USA: Association of Official Analytical Chemists; 2005.
21. Folch, J., Lees, M., Stanley, G.S., 1957. A simple method for the isolation and purification of total lipides from animal tissues. J Biol Chem. 226(1), 497-509. https://doi.org/10.1016/S0021-9258(18)64849-5
22. Garcés, R., Mancha, M., 1993. One-step lipid extraction and fatty acid methyl esters preparation from fresh plant tissues. Anal Biochem. 211(1), 139-143. https://doi.org/10.1006/abio.1993.1244
23. Pfaffl, M.W., 2001. A new mathematical model for relative quantification in real-time RT–PCR. Nucleic Acids Res. 29(9), e45-e45. https://doi.org/10.1093/nar/29.9.e45

24. Vandesompele, J., De Preter, K., Pattyn, F., Poppe, B., Van Roy, N., De Paepe, A., Speleman, F., 2002. Accurate normalization of real-time quantitative RT-PCR data by geometric averaging of multiple internal control genes. Genome Biol. 3(7), research0034-1. https://doi.org/10.1186/gb-2002-3-7-research0034

25. Araújo, B.C., Mata-Sotres, J.A., Viana, M.T., Tinajero, A., Braga, A., 2019. Fish oil-free diets for Pacific white shrimp *Litopenaeus vannamei*: The effects of DHA-EPA supplementation on juvenile growth performance and muscle fatty acid profile. Aquaculture. 511, 734276. https://doi.org/10.1016/j.aquaculture.2019.734276

26. Glencross, B.D., 2009. Exploring the nutritional demand for essential fatty acids by aquaculture species. Rev Aquac. 1(2), 71-124. https://doi.org/10.1111/j.1753-5131.2009.01006.x

27. Suprayudi, M.A., Takeuchi, T., Hamasaki, K., 2004. Effects of Artemia enriched with eicosapentaenoic and docosahexaenoic acid on survival and occurrence of molting failure in megalop larvae of the mud crab *Scylla serrata*. Fish Sci. 70(4), 650-658. https://doi.org/10.1111/j.1444-2906.2004.00853.x

28. Vogt, G., 2019. Functional cytology of the hepatopancreas of decapod crustaceans. J Morphol. 280(9), 1405-1444. https://doi.org/10.1002/jmor.21040

29. Wang, X., Jin, M., Cheng, X., Hu, X., Zhao, M., Yuan, Y., Sun, P., Jiao, L., Betancor, M.B., Tocher, D.R., Zhou, Q., 2021. Dietary DHA/EPA ratio affects growth, tissue fatty acid profiles and expression of genes involved in lipid metabolism in mud crab *Scylla paramamosain* supplied with appropriate n-3 LC-PUFA at two lipid levels. Aquaculture. 532, 736028. https://doi.org/10.1016/j.aquaculture.2020.736028

30. Wang, X., Jin, M., Cheng, X., Luo, J., Jiao, L., Betancor, M.B., Tocher, D.R., Zhou, Q., 2021. Dietary lipid and n-3 long-chain PUFA levels impact growth performance and lipid metabolism of juvenile mud crab, *Scylla paramamosain*. Br J Nutr. 125(8), 876-890. https://doi.org/10.1017/S0007114520003335

31. Stillwell, W., Wassall, S.R., 2003. Docosahexaenoic acid: membrane properties of a unique fatty

acid. Chem Phys Lipids. 126(1), 1-27. https://doi.org/10.1016/S0009-3084(03)00101-4

32. Xu, H., Han, T., Li, X., Wang, J., Zheng, P., Yin, F., Wang, C., 2020. Effects of dietary lipid levels on survival, growth performance, and antioxidant ability of the early juvenile *Scylla paramamosain*. Aquaculture. 528, 735559. https://doi.org/10.1016/j.aquaculture.2020.735559

33. Schlaepfer, I.R., Joshi, M., 2020. CPT1A-mediated fat oxidation, mechanisms, and therapeutic potential. Endocrinology. 161(2), bqz046. https://doi.org/10.1210/endocr/bqz046

34. Olivares-Rubio, H.F., Vega-López, A., 2016. Fatty acid metabolism in fish species as a biomarker for environmental monitoring. Environ Pollut, 218, 297-312. https://doi.org/10.1016/j.envpol.2016.07.005

35. Schwenk, R.W., Holloway, G.P., Luiken, J.J., Bonen, A., Glatz, J.F., 2010. Fatty acid transport across the cell membrane: regulation by fatty acid transporters. Prostaglandins, Leukot Essent Fatty Acids (PLEFA). 82(4-6), 149-154. https://doi.org/10.1016/j.plefa.2010.02.029

36. Wang, Y.C., Chang, P.S., Chen, H.Y., 2007. Tissue expressions of nine genes important to immune defence of the Pacific white shrimp *Litopenaeus vannamei*. Fish Shellfish Immunol. 23(6), 1161-1177. https://doi.org/10.1016/j.fsi.2007.04.004

37. Burge, E.J., Madigan, D.J., Burnett, L.E., Burnett, K.G., 2007. Lysozyme gene expression by hemocytes of Pacific white shrimp, *Litopenaeus vannamei*, after injection with *Vibrio*. Fish Shellfish Immunol. 22(4), 327-339. https://doi.org/10.1016/j.fsi.2006.06.004

38. Miandare, H.K., Mirghaed, A.T., Hosseini, M., Mazloumi, N., Zargar, A., Nazari, S., 2017. Dietary Immunogen® modulated digestive enzyme activity and immune gene expression in *Litopenaeus vannamei* post larvae. Fish Shellfish Immunol. 70, 621-627. https://doi.org/10.1016/j.fsi.2017.09.048

39. Chen, Z., Kong, Y., Huang, Z., Zheng, X., Zheng, Z., Yao, D., Yang, S., Zhang, Y., Aweya, J.J., 2024. Exogenous alpha-linolenic acid and *Vibrio parahaemolyticus* induce EPA and DHA levels mediated by delta-6 desaturase to enhance shrimp immunity. Int J Biol Macromol. 257, 128583. https://doi.org/10.1016/j.ijbiomac.2023.128583

40. Chen, Y.Y., Chen, J.C., Kuo, Y.H., Lin, Y.C., Chang, Y.H., Gong, H.Y., Huang, C.L., 2016. Lipopolysaccharide and β-1, 3-glucan-binding protein (LGBP) bind to seaweed polysaccharides and activate the prophenoloxidase system in white shrimp *Litopenaeus vannamei*. Dev Comp Immunol. 55, 144-151. https://doi.org/10.1016/j.dci.2015.10.023

41. Chen, Y.Y., Chen, J.C., Tayag, C.M., Li, H.F., Putra, D.F., Kuo, Y.H., Bai, J.C., Chang, Y.H., 2016. Spirulina elicits the activation of innate immunity and increases resistance against *Vibrio alginolyticus* in shrimp. Fish Shellfish Immunol. 55, 690-698. https://doi.org/10.1016/j.fsi.2016.06.042

42. Dong, J., Cheng, R., Yang, Y., Zhao, Y., Wu, G., Zhang, R., Zhu, X., Li, L., Li, X., 2018. Effects of dietary taurine on growth, non-specific immunity, anti-oxidative properties and gut immunity in the Chinese mitten crab *Eriocheir sinensis.* Fish Shellfish Immunol. 82, 212-219. https://doi.org/10.1016/j.fsi.2018.08.029

43. Yang, M.J., Xu, D., Yang, D.X., Li, L., Peng, X.X., Chen, Z.G., Li, H., 2020. Malate enhances survival of zebrafish against *Vibrio alginolyticus* infection in the same manner as taurine. Virulence. 11(1), 349-364. https://doi.org/10.1080/21505594.2020.1750123

44. Shankar, R., Murthy, H.S., Sujatha, H.R., Jayaraj, E.G., Tejpal, C.S., Chinthamani, V.S., 2012. Effect of nucleotide on growth, immune responses and resistance of *Macrobrachium rosenbergii* (De Man) to *Macrobrachium rosenbergii nodavirus* (MrNV) and extra small virus (XSV) and *Aeromonas hydrophila* infection. Aquac Int. 20, 1-12. https://doi.org/10.1007/s10499-011-9430-3

45. Xiong, J., Jin, M., Yuan, Y., Luo, J.X., Lu, Y., Zhou, Q.C., Liang, C., Tan, Z.L. 2018. Dietary nucleotide-rich yeast supplementation improves growth, innate immunity and intestinal morphology of Pacific white shrimp (*Litopenaeus vannamei*). Aquac Nutr. 24(5), 1425-1435. https://doi.org/10.1111/anu.12679
