# Supplementary material for: Improvement of growth, lipid metabolism and innate immune response in Pacific white shrimp (Penaeus vannamei) post-larvae through enrichment of live feeds with Schizochytrium sp., taurine and inosine monophosphate
Source: PLoS One. 2026 Mar 5;21(3):e0341999. doi: 10.1371/journal.pone.0341999 (PMC12962509; doi:10.1371/journal.pone.0341999)
Supplement: Attachment — Submitted filename: [Response Letter] PONE-D-25-53314.docx [file pone.0341999.s005.docx]

**Response Letter – ID: PONE-D-25-53314**

Improvement of growth, lipid metabolism and innate immune response in Pacific white shrimp (*Penaeus vannamei*) post-larvae through enrichment of live feeds with *Schizochytrium* sp., taurine and inosine monophosphate

**Dear Editor In-Chief,**

Thank you for the thoughtful suggestions and comments on our manuscript. The authors have carefully revised the manuscript according to the comments/suggestions from reviewers and provided point-by-point response below. I anticipate your positive response and hope that you can find our manuscript suitable for the publication in *PLOS ONE* Journal.

Sincerely yours,

Kyeong-Jun Lee, Ph.D.

**Responses to the comments of Editor**

**1. Please ensure that your manuscript meets PLOS ONE's style requirements, including those for file naming.**

**Answer:** In accordance with the editor’s instructions, all files have been revised to fully comply with the PLOS ONE formatting and file-naming requirements.

**2. In your Methods section, please provide additional information regarding the permits you obtained for the work. Please ensure you have included the full name of the authority that approved the field site access and, if no permits were required, a brief statement explaining why.**

**Answer:** The authority that approved access to the field site was Jeju National University, and this information has been included in the revised manuscript. The corresponding information has been included in the revised manuscript (Lines 468–471).

**3. We note that the grant information you provided in the ‘Funding Information’ and ‘Financial Disclosure’ sections do not match. When you resubmit, please ensure that you provide the correct grant numbers for the awards you received for your study in the ‘Funding Information’ section.**

**Answer:** In accordance with the editor’s instruction, the Funding Information statement has been updated accordingly.

**4. Thank you for stating the following financial disclosure: This work was supported by the CJ Cheiljedang Co., Ltd and Basic Science Research Program through the National Research Foundation of Korea (NRF) funded by the Ministry of Education (RS-2019-NR040078). Please state what role the funders took in the study. If the funders had no role, please state: "The funders had no role in study design, data collection and analysis, decision to publish, or preparation of the manuscript." If this statement is not correct you must amend it as needed. Please include this amended Role of Funder statement in your cover letter; we will change the online submission form on your behalf.**

**Answer:** The funders contributed only by producing and supplying the *Schizochytrium* powder (CJ Cheiljedang Co., Ltd.) and by providing experimental tanks and facilities (National Research Foundation of Korea). The funders had no role in study design, data collection and analysis, decision to publish, or preparation of the manuscript.

**5. Thank you for stating the following in the Acknowledgments Section of your manuscript: This work was supported by the CJ Cheiljedang Co., Ltd and Basic Science Research Program through the National Research Foundation of Korea (NRF) funded by the Ministry of Education (RS472 2019-NR040078). We note that you have provided funding information that is not currently declared in your Funding Statement. However, funding information should not appear in the Acknowledgments section or other areas of your manuscript. We will only publish funding information present in the Funding Statement section of the online submission form. Please remove any funding-related text from the manuscript and let us know how you would like to update your Funding Statement. Currently, your Funding Statement reads as follows: This work was supported by the CJ Cheiljedang Co., Ltd and Basic Science Research Program through the National Research Foundation of Korea (NRF) funded by the Ministry of Education (RS-2019-NR040078). Please include your amended statements within your cover letter; we will change the online submission form on your behalf. Please ensure that your manuscript meets PLOS ONE's style requirements, including those for file naming.**

**Answer:** We mistakenly included funding information in the Acknowledgments section, although it should only appear in the Funding Statement. The funding-related text has now been removed from the Acknowledgments, and the correct Funding Statement has been included in the revised manuscript.

**6. Thank you for stating the following in the Competing Interests section:**

**The authors declare that they have no known competing financial interests or personal relationships that could have appeared to influence the work reported in this paper. Please confirm that this does not alter your adherence to all PLOS ONE policies on sharing data and materials, by including the following statement: "This does not alter our adherence to PLOS ONE policies on sharing data and materials.” (as detailed online in our guide for authors http://journals.plos.org/plosone/s/competing-interests). If there are restrictions on sharing of data and/or materials, please state these. Please note that we cannot proceed with consideration of your article until this information has been declared. Please include your updated Competing Interests statement in your cover letter; we will change the online submission form on your behalf.**

**Answer:** Following the editor’s instructions, we have revised the Competing Interests statement. The updated statement has been added to the revised manuscript.

**7. Thank you for stating the following in the Competing Interests/Financial Disclosure section: The authors declare that they have no known competing financial interests or personal relationships that could have appeared to influence the work reported in this paper. We note that one or more of the authors are employed by a commercial company: CJ Cheiljedang Co. a. Please provide an amended Funding Statement declaring this commercial affiliation, as well as a statement regarding the Role of Funders in your study. If the funding organization did not play a role in the study design, data collection and analysis, decision to publish, or preparation of the manuscript and only provided financial support in the form of authors' salaries and/or research materials, please review your statements relating to the author contributions, and ensure you have specifically and accurately indicated the role(s) that these authors had in your study. You can update author roles in the Author Contributions section of the online submission form. Please also include the following statement within your amended Funding Statement. “The funder provided support in the form of salaries for authors [insert relevant initials], but did not have any additional role in the study design, data collection and analysis, decision to publish, or preparation of the manuscript. The specific roles of these authors are articulated in the ‘author contributions’ section.” If your commercial affiliation did play a role in your study, please state and explain this role within your updated Funding Statement. b. Please also provide an updated Competing Interests Statement declaring this commercial affiliation along with any other relevant declarations relating to employment, consultancy, patents, products in development, or marketed products, etc. Within your Competing Interests Statement, please confirm that this commercial affiliation does not alter your adherence to all PLOS ONE policies on sharing data and materials by including the following statement: "This does not alter our adherence to PLOS ONE policies on sharing data and materials.” (as detailed online in our guide for authors http://journals.plos.org/plosone/s/competing-interests) . If this adherence statement is not accurate and there are restrictions on sharing of data and/or materials, please state these. Please note that we cannot proceed with consideration of your article until this information has been declared. Please include both an updated Funding Statement and Competing Interests Statement in your cover letter. We will change the online submission form on your behalf.**

**Answer:** Among the authors of this manuscript, Young-Mi Lee and Kook-Jin Cho are employed by the commercial company CJ Cheiljedang Co. They were involved in culturing and processing *Schizochytrium* to provide the *Schizochytrium* powder used as the experimental material. The specific roles of these authors are articulated in the “Author Contributions” section. We have updated both the Funding Statement and the Competing Interests Statement accordingly.

**8. If the reviewer comments include a recommendation to cite specific previously published works, please review and evaluate these publications to determine whether they are relevant and should be cited. There is no requirement to cite these works unless the editor has indicated otherwise.**

**Answer:** There were no such comments from the reviewers.

**Responses to the comments of Reviewer #1.**

**1. Conclusion less than enough.**

**Answer:** We appreciate the reviewer’s constructive suggestion. The conclusion section has been revised to provide a more comprehensive summary of the key findings, highlighting the synergistic effects of taurine and IMP supplementation, as well as the practical implications of *Schizochytrium sp.* enrichment for shrimp hatchery nutrition. The revised conclusion also outlines specific future research directions, including the molecular mechanisms and optimal enrichment levels. The updated text appears in the revised manuscript (Lines 441–450).

**2. There are no ethical approval statements.**

**Answer:** We have added a statement regarding ethical approval for animal experimentation, which was granted by Jeju National University. The corresponding information has been included in the revised manuscript (Lines 468–471).

**3. There are no data availability.**

**Answer:** We have included a data availability statement in the revised manuscript (Lines 473–474).

**4. There is no declaration of conflict interests.**

**Answer:** We have added a declaration of competing interests in the revised manuscript (Lines 476–479).

**5. Abstract There are no highlights.**

**Answer:** We have added a “Highlights” section to the revised manuscript (Lines 20–26).

**6. There is no graphical abstract.**

**Answer:** A graphical abstract has been prepared and will be submitted with the revised manuscript.

**7. What is /are the creativity of this work (ordinary study).**

**Answer:** This study provides novel insights into improving the growth, survival, and immune performance of *Penaeus vannamei* during the early post-larval stage, a critical developmental period characterized by high mortality rates. Nutritional enrichment of live feeds for shrimp post-larvae has been insufficiently investigated, despite its potential to enhance larval health and overall aquaculture productivity. Therefore, our work is innovative in that it demonstrates the practical and biological benefits of enriching zoo-live feeds with *Schizochytrium* sp., taurine and inosine monophosphate to improve early-stage shrimp performance. The novelty of this experiment has been described in the introduction (Line 94–100).

**8. Divide the abstract into backgrounds/aims/methods/results and conclusion.**

**Answer:** We thank the reviewer for the valuable suggestion. In response, the abstract has been revised to clearly present the background, aims, methods, results and conclusion in a logical flow.

**9. LN/17—delete /short or running title ?**

**Answer:** According to the journal’s submission guidelines, a short (running) title is required. Therefore, we have retained the running title and revised it to make it clearer and more concise while maintaining the journal’s format (Line 28–29).

**10. LN/20—more details are requested.**

**Answer:** We appreciate the reviewer’s comment. The sentence has been revised to provide more detailed information about the parameters evaluated in this study (Lines 32–35). Specifically, we have clarified that growth performance, lipid metabolism, immune response and survival of *Penaeus vannamei* post-larvae were assessed.

**11. LN/23---which types of nutrients are enriched.**

**Answer:** The term “nutrients” in this sentence refers to *Schizochytrium sp.*, taurine, and inosine monophosphate (IMP), which were used to enrich the live feeds. In contrast, no enrichment additives were applied to the live feeds in the control group. To avoid possible confusion, the term “nutrients” has been replaced with “unenriched rotifers and unenriched *Artemia*” in the revised manuscript (Lines 37, 39).

**12. Huge number of abbreviations are detected—it better to create a separate table for this.**

**Answer:** We agree with the reviewer’s comment. Accordingly, the abbreviations used in the abstract and main text have been clearly defined in the caption of Table 2.

**13. LN/40-41—how ?**

**Answer:** The statement was based on the observed results in this study. Shrimp fed with nutritionally enriched live feeds showed improved growth performance, survival and lipid metabolism. In addition, immune-related genes were upregulated in groups supplemented with taurine and IMP in combination with *Schizochytrium* sp. Therefore, the sentence was written to reflect these findings.

**14. LN/43—add aquaculture industry/gene expression/growth performance /crustacean /immunity to the keywords.**

**Answer:** As suggested by the reviewer, the keywords “aquaculture industry”, “gene expression”, “growth performance”, “crustacean” and “immunity” have been added to the revised manuscript (Line 53–54).

**15. LN/48/76/84—add references.**

**Answer:** We thank the reviewer for the valuable comment. A reference has been added to support the statement (Line 58). The content (Line 84–87) was based on the findings of previous studies [7–14], which were cited to substantiate the expected effects discussed in this section. Similarly, the statement (Line 94–95) was supported by references [15–18], which provide the scientific basis for the study’s objectives and predicted outcomes.

**16. LN/50—on culture water quality—clarify why/how.**

**Answer:** We thank the reviewer for the valuable comment. In the early post-larval stages, the low mobility of larval often results in uneaten micro- or particulate feeds settling at the bottom, which can deteriorate water quality. Numerous studies have reported that live feeds cause less water pollution than formulated diets because they remain suspended longer and are more readily consumed by larvae (Bengtson, 2003; Gonçalves et al., 2024; Melaku et al., 2024).

Bengtson, D.A., 2003. Status of marine aquaculture in relation to live prey: past, present and future. Live feeds in marine aquaculture. pp.1-16.

Goncalves, R., Pfalzgraff, T., & Lund, I. (2024). Impact of live feed substitution with formulated diets on the development, digestive capacity, biochemical composition, and rearing water quality of European lobster (Homarus gammarus, L.) larvae. *Aquaculture*, *586*, 740776.

Melaku, S., Geremew, A., Getahun, A., Mengestou, S., & Belay, A. (2024). Challenges and prospects of using live feed substitutes for larval fish. *Fisheries and Aquatic Sciences*, *27*(8), 475-487.

**17. LN/53—microalgae—for ordinary readers more details are requested.**

**Answer:** We thank the reviewer for the suggestion. The sentence has been revised to provide more detail for general readers, explaining that microalgae are rich in essential fatty acids, particularly DHA and EPA, and serve as effective nutritional sources for enriching live feeds to enhance growth, lipid metabolism and immunity in aquatic animal larvae. The revised sentence appears in the manuscript at Lines 64–67.

**18. LN/60—antioxidant capacity---mechanism?**

**Answer:** Carotenoids such as astaxanthin and β-carotene, as well as vitamins including niacin and pyridoxine, are well-known to enhance antioxidant capacity in shrimp (Zhang et al., 2013; Liu et al., 2024). These bioactive compounds, abundant in *Schizochytrium sp.*, can increase the activities of antioxidant enzymes (e.g., superoxide dismutase, catalase, and glutathione peroxidase), thereby improving overall antioxidant defenses when incorporated into feeds or used to enrich live feeds (Dineshbabu et al., 2019).

Dineshbabu, G., Goswami, G., Kumar, R., Sinha, A., Das, D., 2019. Microalgae–nutritious, sustainable aqua-and animal feed source. J Funct Foods. 62, 103545.

Liu, A., Dumas, A., Hernandez, J.M., Santigosa, E., 2024. Vitamin nutrition in shrimp aquaculture: A review focusing on the last decade. Aquaculture, 578, 740004.

Zhang, J., Liu, Y.J., Tian, L.X., Yang, H.J., Liang, G.Y., Yue, Y.R., Xu, D.H., 2013. Effects of dietary astaxanthin on growth, antioxidant capacity and gene expression in Pacific white shrimp *Litopenaeus vannamei*. Aquac Nutr, 19(6), 917-927.

**19. Introduction is extremely long and repeated, Rewrite it again, Aims should be more clarified, Novelty should be more concentrated.**

**Answer:** We appreciate the reviewer’s insightful comments. The Introduction has been revised to remove repetitive content and improve logical flow while maintaining essential background information. The research aims have been clarified, and the novelty of this study—specifically the first evaluation of combined *Schizochytrium* sp., taurine, and inosine monophosphate enrichment in live feeds for *Penaeus vannamei*—has been emphasized in the final paragraph. The revised section appears at Lines 94–100 in the manuscript.

**20. Materials and Methods LN/94-101—add reference and what about the physical and chemical nature of these materials.**

**Answer:** We thank the reviewer for the helpful comment. The source and characteristics of the *Schizochytrium* powder used in this study have been clarified in the revised manuscript (Lines 102–113). The *Schizochytrium* powder was supplied by CJ CheilJedang Corp. (Seoul, South Korea), and its production method is described in the corresponding Korean patent (KR 20240026429A; Lee et al., 2024).

**21. LN/104-108—delete.**

**Answer:** We appreciate the reviewer’s suggestion. That abbreviation (previously Line 48) has been deleted as recommended. However, the content in Lines 116–117 provides essential information regarding the characteristics of the materials. Removing these lines would hinder the clarity and interpretation of the experimental setup; therefore, they have been retained in the revised manuscript.

**22. Table -1—should be directed toward the results.**

**Answer:** We appreciate the reviewer’s suggestion. However, Table 1 presents the proximate composition and fatty acid profile of the experimental materials used in this study. Therefore, it is considered more appropriate to include it in the Materials and Methods section rather than in the Results, as it describes the characteristics of the feed ingredients rather than experimental outcomes.

**23. Figure(1&2)with the results**

**Answer:** We thank the reviewer for the comment. Figs 1 and 2 do not depict experimental outcomes; rather, they illustrate the effectiveness of nutritional enrichment in the live feeds and demonstrate that the feeds are suitable for use in the subsequent shrimp rearing experiments. Therefore, we consider it more appropriate to include these figures in the Materials and Methods section to emphasize the characteristics of the feed materials rather than in the Results section.

**24. The whole experimental protocol has done according to whom.**

**Answer:** Thank you for your comment. The overall experimental protocol was established by integrating and adapting methodological procedures reported in previous studies to optimize the conditions for our experimental environment. Specifically, the protocols for live feed enrichment, larval rearing, and analytic procedures were referenced and modified based on the following studies: Jamali et al. (2015), Silva et al. (2021), Tiong et al. (2020), and Kim et al. (2024). These studies collectively guided the preparation of enriched rotifers and Artemia, the rearing conditions for *Penaeus vannamei* post-larvae, and the evaluation of growth, immunity and physiological responses under controlled laboratory conditions.

Jamali, H., Imani, A., Abdollahi, D., Roozbehfar, R., & Isari, A. (2015). Use of probiotic Bacillus spp. in rotifer (*Brachionus plicatilis*) and Artemia (*Artemia urmiana*) enrichment: Effects on growth and survival of Pacific white shrimp, *Litopenaeus vannamei*, Larvae. Probiotics and antimicrobial proteins, 7(2), 118-125.

Silva, D. A., de Lima, P. C. M., da Silva, A. E. M., de Oliveira Filho, P. R. C., da Silva, S. M. B. C., Olivera Galvez, A., & Brito, L. O. (2021). Effects of adding rotifers on the water quality, plankton composition and growth of Pacific white shrimp, *Litopenaeus vannamei* juvenile, when cultured with biofloc technology. Aquaculture Research, 52(9), 4380-4393.

Tiong, I. K. R., Nagappan, T., Wahid, M. E. A., Muhammad, T. S. T., Tatsuki, T., Satyantini, W. H., ... & Sung, Y. Y. (2020). Antioxidant capacity of five microalgae species and their effect on heat shock protein 70 expression in the brine shrimp Artemia. Aquaculture Reports, 18, 100433.

Kim, S., Shin, J., Lim, H., Ko, D., Eom, G., Lim, J., ... & Lee, K. J. (2024). Effects of feeding poly-β-hydroxybutyrate enriched Artemia nauplii on the growth, digestibility and immunity of Pacific white shrimp (*Penaeus vannamei*) post-larvae. Fisheries and Aquatic Sciences, 27(7), 447-455.

**25. LN/176-178---2 different styles of writing references were detected—why ? same style should be.**

**Answer:** We thank the reviewer for the comment. The references in Lines 192–194 have been revised to follow the journal’s required style, ensuring consistent formatting throughout the manuscript.

**26. LN/186-189—why you did not do a histological section for more confirmation.**

**Answer:** We thank the reviewer for this suggestion. In this study, we prioritized qRT-PCR and fatty acid analyses, which were considered more critical for evaluating the effects of the enrichment. Consequently, the number of hepatopancreas samples available for histological analysis was limited. In addition, the small size of the post-larval shrimp made the hepatopancreas tissues too small for reliable histological sectioning.

**27. LN/214—there is no reference for the statistical analysis**

**Answer:** Thank you for your comment. A reference for the statistical analysis has been added, and the information has been incorporated in Line 234.

**28. There is no plan for the study area.**

**Answer:** We thank the reviewer for the comment. The feeding trials were conducted at the Kidang Marine Science Institute, Jeju National University, Jeju, Korea. This information has been added to the manuscript at Lines 165–167.

**29. There is no IACUC-code –should be.**

**Answer:** We thank the reviewer for pointing this out. This study was approved by the Jeju National University Institutional Animal Care and Use Committee (IACUC), and the corresponding approval information has been added to the manuscript at Lines 468–471.

**30. Results What about the clinical signs of the examined shrimp.**

**Answer:** We appreciate the reviewer’s comment. No abnormal clinical signs were observed in any of the experimental shrimp. In both feeding trials, the growth performance and survival rate were within the normal range for *P. vannamei*, indicating that the shrimp exhibited no health- or behavior-related abnormalities during the experimental period.

**31. What about the PM changes (gross figures are needed).**

**Answer:** We appreciate the reviewer’s comment. Photographs showing the gross morphological appearance of shrimp after the feeding trials have been added to the Supporting Information. No abnormal external signs were observed, including discoloration of the shell, hepatopancreas color changes, melanization, body-wall damage, deformities, or abnormal pigmentation.

**32. What about the mortality percent.**

**Answer:** We appreciate the reviewer’s comment. The survival data representing mortality outcomes for both feeding trials are provided in Tables 5 and 6.

**33. LN/240---why did you put this table with results not at M&M.**

**Answer:** We appreciate the reviewer’s comment. We have already described this table in the Materials and Methods section (Lines 187–194). The nutritional composition and fatty acid profiles of the enriched live feeds play an essential data in the feeding trials.

**34. LN/241-247/250-256---be more summarized.**

**Answer:** We appreciate the reviewer’s suggestion. We have removed unnecessary descriptions and revised the sentences in all Table caption to be more concise and focused.

**35. 8 tables and 6 figures—recheck more than enough.**

**Answer:** We thank the reviewer for this comment. We carefully re-evaluated all tables and figures and confirmed that each item presents essential data directly supporting the study objectives. Nevertheless, we reduced unnecessary descriptions, condensed overly detailed explanations, and ensured that no redundant information remains. Thus, all tables and figures now represent only the core results required for proper interpretation of the study.

**36. It is extremely very long. Rewrite it again.**

**Answer:** We appreciate the reviewer’s comment. In response, we thoroughly revised the Results section by removing unnecessary details, eliminating redundancies, and condensing descriptions wherever possible. The revised version is now substantially more concise while retaining all essential findings required for accurate interpretation.

**37. Discussion It is very long –be more concise.**

**Answer:** We thank the reviewer for the valuable comment. We have removed redundant and non-essential content from the Discussion and revised the text to be more concise and focused.

**38. References - Some cited references need to be more updated.**

**Answer:** We appreciate the reviewer’s comment. Some older references have been updated with more recent publications to reflect the latest findings.

**39. Some cited references with missing data –recheck.**

**Answer:** We appreciate the reviewer’s comment. All references have been checked for missing information, and necessary corrections have been made.

**40. Huge number of references were used–why.**

**Answer:** We appreciate the reviewer’s comment. A large number of references were necessary to provide a comprehensive and scientific interpretation of various ingredients, including *Schizochytrium*, taurine and IMP, and the multiple analyzed parameters. All references have been carefully reviewed to remove any unnecessary or inappropriate citations.

**41. Some journal names were written abbreviated, while others were not—why ? same style should be.**

**Answer:** We appreciate the reviewer’s comment. All journal names have been standardized to their abbreviated forms in accordance with the journal’s reference style.

**42. As volume/issue/pages/number—available—so no need for the link(s)—apply for all.**

**Answer:** We appreciate the reviewer’s comment. All DOI or URL links have been removed, and references now include only volume, issue, and page numbers as appropriate, in accordance with the journal’s guidelines.

**43. Some cited references contained more than 6 authors—why—should be 6 at the maximum plus et al with the last ones---apply for all.**

**Answer:** We appreciate the reviewer’s comment. All references with more than six authors have been revised to list the first six authors followed by “et al.” in accordance with the journal’s guidelines.

**44. All references should be rewritten.**

**Answer:** We appreciate the reviewer’s comment. All references have been carefully revised and corrected in accordance with the journal’s guidelines.

**45. There are no gross figures—why.**

**Answer:** We appreciate the reviewer’s comment. Photographs showing the gross morphological appearance of shrimp after the feeding trials have been added to the Supporting Information. No abnormal external signs were observed, including discoloration of the shell, hepatopancreas color changes, melanization, body-wall damage, deformities, or abnormal pigmentation.

**Responses to the comments of Reviewer #2.**

**1. Line 24 – and.**

**Answer:** It has been corrected to 'and'.

**2. Lines 46-47 – Delete.**

**Answer:** The sentence has been deleted.

**3. Line 100 – reference.**

**Answer:** We appreciate the reviewer’s comment. We have added a reference describing the manufacturing process of *Schizochytrium* powder (Line 102–113, [19]), and the references for the proximate composition and fatty acid profile have been included in Lines 187–194.

**4. Line 139 – replicates of each group did not mention.**

**Answer:** We appreciate the reviewer’s comment. Each experimental group was conducted in quadruplicate, and this information has been added to the manuscript (Line 130, 151–152).

**5. Line 156 – material of tank.**

**Answer:** The tanks were made of acrylic and had a rectangular shape with a capacity of 96 L (Line 168, 178).

**6. Line 159 – methodology of water quality (mention).**

**Answer:** We appreciate the reviewer’s comment. Dissolved oxygen, temperature, pH, salinity and ammonia were measured three times daily using a ProQuatro Multiparameter Meter (YSI, Yellow Springs, OH, USA), and this information has been added to the manuscript (Line 170–172, 179–181).

**7. Line 223 – table did not show P-value.**

**Answer:** We appreciate the reviewer’s comment. Due to the large number of parameters presented in the table, it was not feasible to include individual P-values for each. Instead, we have clarified in the table legend that different superscripts in the same row indicate significant differences (*P* < 0.05).
